# Identification of Synergistic, Clinically Achievable, Combination Therapies for Osteosarcoma

**DOI:** 10.1038/srep16991

**Published:** 2015-11-25

**Authors:** Diana Yu, Elliot Kahen, Christopher L. Cubitt, Jeremy McGuire, Jenny Kreahling, Jae Lee, Soner Altiok, Conor C. Lynch, Daniel M. Sullivan, Damon R. Reed

**Affiliations:** 1 Sunshine Lab, H. Lee Moffitt Cancer Center and Research Institute, Tampa, FL 33612 Florida USA; 2 Translational Research Lab, H. Lee Moffitt Cancer Center and Research Institute, Tampa, FL 33612 Florida USA; 3 Sarcoma Department, H. Lee Moffitt Cancer Center and Research Institute, Tampa, FL 33612 Florida USA; 4 Chemical Biology and Molecular Medicine Program, H. Lee Moffitt Cancer Center and Research Institute, Tampa, FL 33612 Florida USA; 5 Adolescent and Young Adult Program, H. Lee Moffitt Cancer Center and Research Institute, Tampa, FL 33612 Florida USA; 6 Department of Biostatistics and Bioinformatics, H. Lee Moffitt Cancer Center and Research Institute, Tampa, FL 33612 Florida USA; 7 Department of Anatomic Pathology, H. Lee Moffitt Cancer Center and Research Institute, Tampa, FL 33612 Florida USA; 8 Department of Tumor Biology, H. Lee Moffitt Cancer Center and Research Institute, Tampa, FL 33612 Florida USA; 9 Department of Blood and Marrow Transplantation, H. Lee Moffitt Cancer Center and Research Institute, Tampa, FL 33612 Florida USA

## Abstract

Systemic therapy has improved osteosarcoma event-free and overall survival, but 30–50% of patients originally diagnosed will have progressive or recurrent disease, which is difficult to cure. Osteosarcoma has a complex karyotype, with loss of p53 in the vast majority of cases and an absence of recurrent, targetable pathways. In this study, we explored 54 agents that are clinically approved for other oncologic indications, agents in active clinical development, and others with promising preclinical data in osteosarcoma at clinically achievable concentrations in 5 osteosarcoma cell lines. We found significant single-agent activity of multiple agents and tested 10 drugs in all permutations of two-drug combinations to define synergistic combinations by Chou and Talalay analysis. We then evaluated order of addition to choose the combinations that may be best to translate to the clinic. We conclude that the repurposing of chemotherapeutics in osteosarcoma by using an *in vitro* system may define novel drug combinations with significant *in vivo* activity. In particular, combinations of proteasome inhibitors with histone deacetylase inhibitors and ixabepilone and MK1775 demonstrated excellent activity in our assays.

Over the past few decades, there has been little progress in terms of developing more effective chemotherapies for osteosarcoma. This is true despite diligent efforts to explore many agents through collaborative trials that have included agents such as trastuzumab, interferon alfa-2b, ifosfamide, etoposide, zoledronic acid, and MTP-PE^1^,^2^,^3^,^4^,^5^. Current “standard of care” pediatric osteosarcoma therapy consists of three agents: high-dose methotrexate, doxorubicin, and cisplatin, with the former two being FDA-approved for this indication. Data suggest that this combination is the most effective for young adults as well, but 10-year event-free survival rates for this population are 5–10% lower than the roughly 65% pediatric rate^6^,^7^,^8^. Older patients are typically treated with these same agents or given a combination of doxorubicin and cisplatin, with occasional use of ifosfamide^9^. Due to osteosarcoma’s rarity, clinical trials are difficult and time consuming to conduct, increasing the need for strong preclinical data to inform clinical trials. Meanwhile, many agents have been FDA-approved for adult carcinomas that cannot all be evaluated clinically for use in osteosarcoma^10^.

There have been numerous preclinical efforts to better understand the pathophysiology of osteosarcoma and test agents with diverse mechanisms of actions on osteosarcoma models in order to inform future trials, including some of our own work with cell cycle inhibitors^11^,^12^,^13^,^14^,^15^. Furthermore, osteosarcoma occurs spontaneously in many animal species including canines where the biology, therapy and response are similar to humans^16^,^17^,^18^. Notwithstanding these efforts, there is not an obvious agent with sufficient activity to explore prospectively in frontline clinical trials at this time^19^,^20^. Sequencing of osteosarcoma tumors has demonstrated that osteosarcoma biology seems to rely on dysfunctional p53 in virtually all clinical cases with frequent translocations in intron 1 of the TP53 gene^21^. This genomic analysis revealed significant tumor-to-tumor variability through varied and numerous structural variations. As a result, a consistent therapeutic target has proven to be elusive. Despite tumor variability, we hypothesize that p53 plays a significant role in osteosarcoma tumorigenesis. For this study, we selected well-characterized cell lines that demonstrate p53 inactivation as our models. Both SAOS-2 and MG-63 have disruptions in intron 1 of TP53^22^. HOS and 143B cells are derived from the same patient and share an inactivating TP53 point mutation at position (R156P)^23^. U2OS is TP53 wildtype but contains an amplification of MDM2 rendering p53 hypofunctional^24^.

We set out to develop a system to evaluate combinations of many agents that can then be rapidly translated into clinical trials in a clinically relevant manner. The methodology was optimized to incorporate past lessons learned from *in vitro* experiments that did not translate well into clinic. This was at least in part due to studied drug concentrations that were not achievable or lengths of exposure not possible as a result of metabolism^25^,^26^. By using largely FDA-approved agents, agents studied in pediatric trials^27^, and agents with strong preliminary data for an osteosarcoma subtype, we anticipated that we could efficiently develop strong preclinical data to help inform clinical trials in osteosarcoma. All steps and experiments for combination therapy were developed and conducted in the context of the eventual clinical trial. This included careful exploration of current and previously evaluated clinical schedules that have been tolerable, demonstrated non-overlapping toxicities, included pharmacokinetic data and cytochrome P450 metabolism, and described other metabolic details that would avoid obvious drug-drug interactions.

## Results

### Single-agent activity at clinically achievable levels and durations

**Figure 1:**
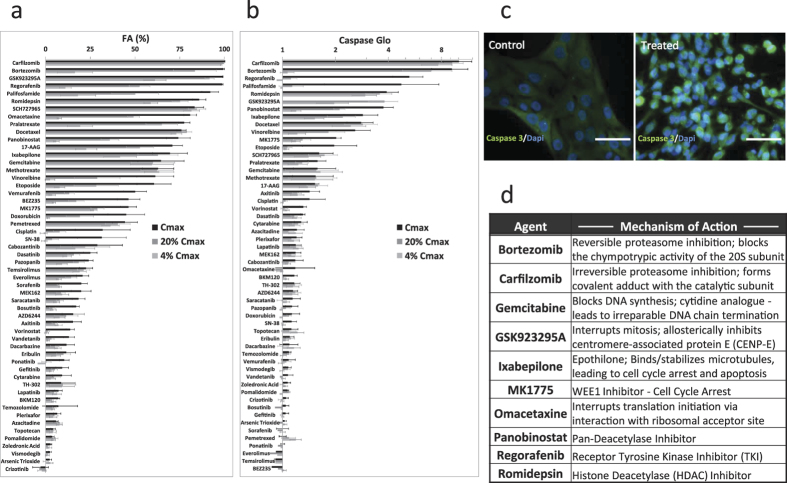
Single-agent activities of 54 therapeutic compounds screened against 5 osteosarcoma cell lines. (**a**) Percent reduction in CellTiter-Glo signal indicating decreased ATP production at 72 hours post-drug treatment (normalized to vehicle controls). (**b**) Relative fold change in Caspase-Glo signal indicating cell death 24 hours post-drug treatment (normalized to vehicle controls). (**c**) Immunocytochemistry staining of caspase-3 activity in MG63 cells after 6-hour treatment with GSK923295A (7122 ng/mL) showing caspase-3 up-regulation in response to drug treatment. (**d**) Top 10 drug candidates with diverse mechanisms-of-action were selected from single-agent screening results to be further evaluated in 45 drug combinations for synergistic effects against pediatric osteosarcoma. N = 3 biological replicates. Scale bar: 20 μm.

We first characterized the single-agent activity of a panel of 54 therapeutic candidates (Supplemental Table S1) using 5 pediatric osteosarcoma cell lines (143B, MNNG/HOS, MG63, U2OS, Saos2). Single-agent anti-tumor activities were assessed at C_max_, 20% C_max_, and 4% C_max_ using Caspase-Glo and CellTiter-Glo luminescence assays at 24 and 72 hours to indicate cell metabolism and apoptosis, respectively. Our screening results using the two assays provided collaborating evidence to indicate the most efficacious agents for the osteosarcoma cells at the tested concentrations (Fig. 1a,b). Interestingly, we obtained low Caspase-Glo signal for GSK923295A at the highest tested concentration but not at the lower concentrations. We hypothesized that the high level of GSK923295A induced a fast onset of apoptosis that could not be detected by Caspase-Glo at 24 hours due to degradation of the caspase enzyme. To verify this, immunohistochemistry was performed on osteosarcoma cells after 6-hour treatment with GSK923295A. We confirmed cell apoptosis due to the drug by demonstrating an up-regulation of caspase-3 activity relative to the vehicle controls (Fig. 1c).

Six agents that achieved >80% anti-tumor activity by CellTiter-Glo assay and >1.5-fold increase in caspase-3/7 activation were selected to move forward to combinatorial screening for potential synergistic effects (Fig. 1d). Additionally, gemcitabine, panobinostat, MK1775, and ixebepilone were selected based on promising activity as well as to expand the diversity of mechanisms of action for combinatorial testing.

### Evaluation for active and synergistic combinations

**Figure 2:**
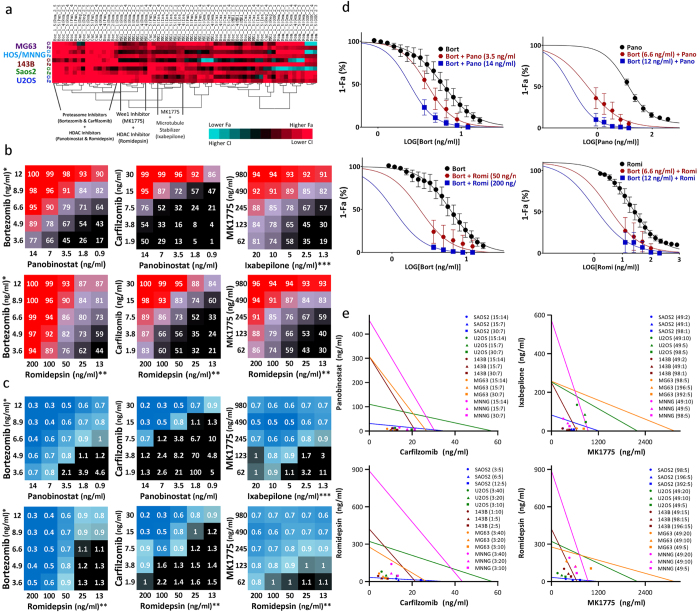
Combination screening results. (**a**) clustering results showing top combination picks based on FA. (**b**) FA of the top 6 combinations evaluated in 5 osteosarcoma cell lines using CT-Glo viability assay. (**c**) CI of the top 6 combinations evaluated in 5 osteosarcoma cell lines using non-constant ratio CI method. (**d**) single and combination drug-response curves for bortezomib+panobinostat and bortezomib + romidepsin combinations. (**e**), isobologram analysis of carfilzomib + panobinostat, carfilzomib + romidepsin, MK1775 + ixabepilone, and MK1775 + romidepsin at ED90. *Alternative bortezomib concentrations used with MG63 (5.00, 3.70, 2.74, 2.03, 1.50 ng/mL). **Alternative romidepsin concentrations used with 143B (150, 75.0, 37.5, 18.8, 9.40 ng/mL); Saos2 (25.0, 12.5, 6.25, 3.13, 1.56 ng/mL). ***Alternative ixabepilone concentrations used with U2OS; MNNG/HOS (100, 50.0, 25.0, 12.5, 6.25 ng/mL); MG63 (25.0, 12.5, 6.25, 3.13, 1.56 ng/mL). The exact drug concentrations used are summarized in Supplemental Table S3.

We used a 5 × 5 checker-board matrix format that assessed each of the 10 active agents at 5 clinically achievable concentrations and multiple drug ratios to identify synergy (Supplemental Figure S1A). Full dose-response curves were obtained for the top 10 drug panel, and the CI values for all 45 combinations were calculated using CalcuSyn 2.0 and custom-designed analysis package based on the Chou and Talalay method (Supplemental Figure S1C). FA and CI values of all combinations that yielded FA > 0.85 in all 5 osteosarcoma cell lines are summarized in Supplemental Table S2. Our combination screening identified several promising drug combinations for the treatment of pediatric osteosarcoma, which were sorted by cluster analysis (Fig. 2a). Of these, we narrowed the results to 6 top combinations that produced >90% anti-tumor activity (Fig. 2b) while demonstrating strong synergy: carfilzomib:panobinostat, carfilzomib:romidepsin, bortezomib:panobinostat, bortezomib:romidepsin, MK1775:ixabepilone, and MK1775:romidpesin (Fig. 2c). Due to the steep sigmoidal drug-response curve observed for bortezomib, it was assessed at 5 concentrations with a dilution factor of 1:1.35, whereas romidepsin and panobinostat were assessed at five concentrations with a dilution of 1:2. Potentiation in bortezomib combinations was determined by fitting the dose-response relationships of single agents and drug combinations to a sigmoidal four-parameter logistic curve on a semi-log plot (Fig. 2d). Addition of 14 ng/mL panobinostat potentiated the cytotoxic effects of bortezomib by shifting the IC50 of bortezomib ~1.7-fold, from 7.45 to 4.35 ng/mL, whereas addition of 6.59 and 12 ng/mL bortezomib potentiated the cytotoxic effects of panobinostat by shifting the IC50 of panobinostat ~4.4-fold and ~4.8-fold, from 11.85 to 2.7 ng/mL and 2.5 ng/mL, respectively. Similarly, the addition of 50 and 200 ng/mL romidepsin potentiated the cytotoxicity of bortezomib by ~1.6-fold and ~2.5-fold, respectively, whereas addition of 6.59 and 12 ng/mL bortezomib potentiated the effects of romidepsin by ~4.1-fold and 6.8-fold, respectively (Fig. 2d, Supplemental Table S4). Isobologram representations for carfilzomib:panobinostat, carfilzomib:romidepsin, MK1775:ixabepilone, and MK1775:romidepsin combinations at constant drug molar ratios further demonstrated the synergistic effects of combining these agents in clinically achievable dose ranges (Fig. 2e). Moreover, we utilized the Mixlow method of synergy calculation on data points at constant molar ratios for these 4 combinations to further verify that synergy is observed at high FA levels (Supplemental Figure S2). We consistently observed strong activity and synergistic effects when combining the HDAC inhibitors romidepsin and panobinostat with the proteasome inhibitors bortezomib and carfilzomib or the cell cycle inhibitor MK1775 (Fig. 2a). In addition, we observed promising synergy between MK1775 and ixabepilone, a microtubule inhibitor.

### Order-of-addition analysis of combinations of interest

**Figure 3:**
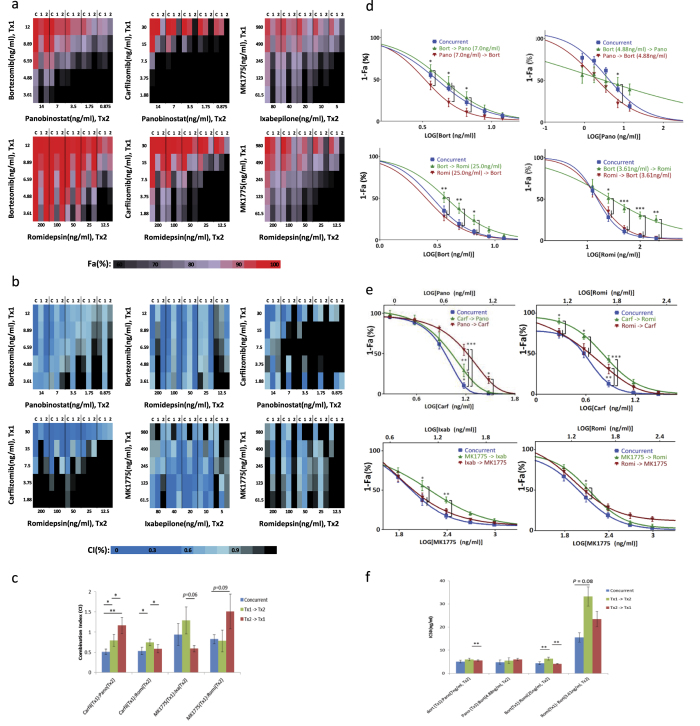
Order-of-addition analysis of the top 6 two-drug combinations assessed in 5 osteosarcoma cell lines FA (a) and CI (b) results using CT-Glo viability assay and non-constant ratio CI method, respectively. *C* indicates concurrent treatment, 1: Tx1 given 24 hours prior to T × 2, 2: T × 2 given 24 hours prior to T × 1. (**c**) combination indices of carfilzomib:romidepsin, carfilzomib:panobinostat, MK1775:romidepsin, MK1775:ixabepilone in panel E averaged across ED80, ED85, ED90, ED95. (**d**) combination drug-response curves of bortezomib:romidepsin (top) and bortezomib:panobinostat (bottom) reported by CT-Glo. (**e**) combination drug-response curves of carfilzomib:romidepsin (3:20), carfilzomib-panobinostat (30:14), MK1775:romidepsin (98:20), MK1775:ixabepilone (98:8) reported by CT-Glo. (**f**) IC50 values of bortezomib:panobinostat and bortezomib:romidepsin combinations in panel A. Statistical comparison used two-way ANOVA with post-hoc Tukey test (**d,e**) and 2-tail paired *t*-test (**c,f**). *P < 0.05, **P < 0.01, ***P < 0.001).

To determine whether the combinatorial effects were dependent on treatment order, we next evaluated the order of addition in these 6 efficacious two-drug combinations using the 5 × 5 checker-board matrix format at 5 clinically achievable concentrations (Fig. 3a,b). A comparison of different orders of drug application and concurrent drug application showed slight variations in the FA and CI values between the osteosarcoma cell lines; however, the combinations continued to demonstrate synergy with high FA (Table 1, Supplemental Table S5). At the highest tested concentrations, bortezomib and romidepsin demonstrated similar FA levels; however, concurrent application and treatment with romidepsin 24 hours prior to bortezomib produced slightly lower CI, suggesting better synergy (Table 1, Fig. 3a,b). This is further demonstrated at additional drug ratios where treatment with bortezomib prior to romidepsin resulted in a right shift in the drug-response curve (Fig. 3d) and significant increase in the IC50 (Fig. 3f). A similar trend was observed for bortezomib and panobinostat at the highest tested levels, where concurrent application and treatment with the HDAC inhibitor prior to bortezomib produced slightly better synergy (Table 1). Drug-response plots of additional concentration ratios showed a left shift when panobinostat was given prior to bortezomib (Fig. 3d) and a decrease in the IC50 (Fig. 3f). Nevertheless, since potentiation was only observed when bortezomib followed panobinostat, but not when panobinostat followed bortezomib, the differences between the orders of addition in this case may be nominal. Interestingly, for panobinostat and carfilzomib, a proteasome inhibitor similar to bortezomib, the best synergy was achieved with concurrent application of the 2 drugs, at both the highest tested levels (Table 1) and for the drug ratio 30:14 (carfilzomib:panobinostat) (Fig. 3f), with a significantly lower CI value (Fig. 3c). Moreover, we found a pattern for carfilzomib and romidepsin similar to that of bortezomib and romidepsin (Fig. 3c,e), indicating that the 2 proteasome inhibitors may elicit similar intracellular events when combined with Romidepsin, and we noted that the presence of romidepsin, either concurrently or prior to proteasome inhibition, may be important for enhanced synergy. In contrast to the preferred order of addition of romidepsin prior to or concurrent with the proteosome inhibitors, we found a trend of reduced efficacy and synergy when romidepsin was applied before MK1775 (Table 1, Fig. 3c,e). Finally, varying the order of addition of MK1775 and ixabepilone demonstrated slight improvement in synergy when ixabepilone was applied before MK1775 (Fig. 3c,e).

### Cytotoxicity of combinations of interest in an *ex vivo* xenograft and in normal cells

These top 6 combinations were also studied in an ex-vivo assay from a murine xenograft derived from a 50-year-old patient who had unresectable, metastatic osteosarcoma at diagnosis that progressed through multi-agent chemotherapy. Excellent efficacy was seen in 5 of the 6 combinations, and synergy was observed for all 6 (Table 2). Interestingly, although synergistic, reduced efficacy was observed for MK1775 and ixabepilone. This could potentially be due to the reduced levels of MK1775 used compared with the cell line experiments.

**Figure 4:**
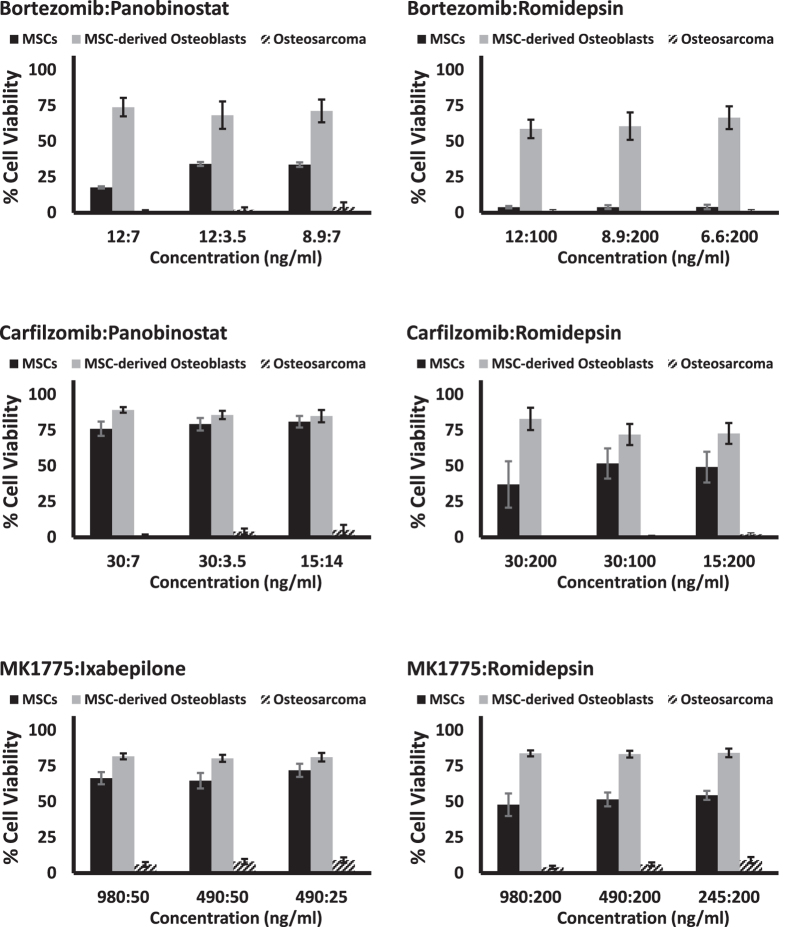
Cytotoxicity of top 6 combinations evaluated in primary MSCs and primary MSC-derived osteoblasts in comparison with osteosarcoma cell lines.

To determine the toxicity of our top candidates against similar but non-tumor cell types, we tested the cytotoxicity of the combinations using primary MSCs and MSC-derived osteoblasts to assess the sensitivity of non-cancer cells to our drug combinations. Strikingly, our combinations of interest showed reduced toxicity in primary MSCs and MSC-derived osteoblasts compared to the osteosarcoma cells (Fig. 4). Furthermore, the MSC-derived osteoblasts appeared to have higher tolerance than the MSCs, suggesting that the drug combinations may preferentially affect proliferating cells over quiescent cell populations

## Discussion

In this study, we tested 54 clinically utilized agents with varied mechanisms of action to better prioritize these agents for potential clinical trials or additional preclinical testing in osteosarcoma. This was done in a relatively high-throughput fashion to determine synergistic combinations of chemotherapies for osteosarcoma, and we report multiple 2-drug combinations with impressive *in vitro* activity. We also tested combinations of targeted agents and found multiple combinations that demonstrated synergy in our tested cell line models. We consistently observed that HDAC and proteasome inhibitor combinations demonstrated good activity. The Wee1 inhibitor MK1775 was also particularly active with romidepsin or ixabepilone.

Both HDAC and proteasome inhibitors have mechanisms of action that result in multiple effects on cellular processes^28^,^29^,^30^,^31^. Both have been implicated in cell cycle disruption and lead to apoptosis in multiple models^28^,^29^,^32^,^33^. The antitumor effects of HDAC inhibitors may be related to epigenetic effects on DNA and resultant transcriptional modification of many genes including perhaps a common gene set in malignant cells^32^. HDACs have numerous other roles in the cell, perhaps through epigenetic modification but also through an increasingly clear effect on many other proteins that are modified or interact with HDACs^29^,^31^. These include roles in cell cycle, reactive oxygen species production, apoptosis, immunomodulatory effects, angiogenesis, and tumor metastasis^29^. Our tested HDAC inhibitors have broad spectrum activity, inhibiting class I and II HDACs (panobinostat and vorinostat), although romidepsin demonstrates some selectivity toward class I HDACs 1 and 2^29^,^31^.

Investigations of HDAC inhibitors in canine osteosarcoma cells have proposed perturbations in the oxidative phosphorylation, cytoskeleton remodeling, cell cycle, and ubiquitin-proteasome pathways by gene expression analysis^34^. Another investigation of a drug-resistant cell line treated with both an HDAC and a DNA methyltransferase inhibitor pointed to apoptosis and differentiation pathways as key mechanisms of action^35^. Others have proposed a miRNA mechanism through c-MYC, again requiring DNA methytransferase inhibitors for an effect^36^. The HDAC inhibitor valproic acid, not active by our methods, has sensitized canine osteosarcoma to doxorubicin^34^.

The proteasome inhibitors that we investigated, bortezomib and carfilzomib, have a clearer target of action, namely the 20S subunit of the 26S proteasome, than the HDAC inhibitors, which inhibit a process involving a plethora of different cellular proteins. The effects of inhibiting the proteasome are many, however, with aggregation of numerous proteins from a multitude of pathways usually resulting in cellular dysfunction and often leading to apoptosis. Some of these cellular processes include angiogenesis, the cell cycle, the unfolded protein response, metastasis, and tumor-stromal interactions^28^,^37^. Intriguingly, one study has shown that proteasome inhibition with bortezomib leads to osteoblast differentiation and increased bone formation^38^. This has also been observed clinically, where bortezomib activity is associated with osteoblast activation, especially in myeloma patients who have a rapid response^39^. Others have explored proteasome inhibitors in osteosarcoma and have proposed as potential mechanisms increased MAPK pathway activity and apoptosis^40^, decreased invasion and increased G2M cell cycle arrest^41^, and increased apopotosis through RUNX2 stabilization^42^. Pathologically, osteosarcomas are characterized by observable osteoid production. Osteoid is largely composed of type I collagen, a highly expressed protein in osteosarcomas^43^. This focus on protein production and continual endoplasmic reticular stress may underlie osteosarcoma sensitivity to proteasome inhibition (personal communication, Paul Meltzer).

Because it has been suggested that HDAC inhibitors inhibit the proteasome pathway^34^ and proteasome inhibition decreases HDAC inhibitor levels^44^, this combination has a biologic rationale for synergy^37^. This combination has also been clinically studied in multiple myeloma, along with dexamethasone, with good tolerability and effect at doses and a schedule similar to single-agent use^45^. The most commonly observed side effects were hematologic, including thrombocytopenia, along with neuropathic effects and fatigue. Panobinostat has recently received FDA approval for this indication following a trial with panobinostat and bortezomib in patients with multiple myeloma (NCT01023308). A completed study with bortezomib and vorinostat has demonstrated safety in the pediatric population, with toxicities similar to adult studies (i.e., predominantly neuropathic and hematologic)^46^. A single osteosarcoma patient was treated on this trial without a response.

The Wee1 inhibitor MK1775 has been demonstrated to have effects on sarcomas, including osteosarcoma^12^,^13^,^15^. While inhibition of Wee1 initially seemed straightforward mechanistically, it has become more apparent that this protein’s function includes coordination of the cell cycle via modification of histones, along with replication fork transcription initiation during S phase^47^,^48^. Thus, HDAC and proteasome inhibitors and the MK1775 inhibitor share cell cycle disruption as a mechanism, perhaps suggesting that osteosarcoma is remarkably vulnerable to this particular perturbation. In combination, the synergism of MK1775 and romidepsin may stem from manipulation of the cell cycle at the G2/M checkpoint by MK1775, thereby enhancing the lethality of romidepsin possibly through preventing DNA repair mechanisms^49^.

We observed a range of activity among the microtubule agents, which either stabilized or inhibited polymerization of beta-tubulin during mitosis. Among members of this class of drugs, which have consistently demonstrated some effectiveness in the Pediatric Preclinical Testing Program, we determined that ixabepilone had the best activity across cell lines^11^,^50^,^51^. A phase II study of ixabepilone based on murine xenograft work included 11 osteosarcoma patients without any responses and only a single patient receiving more than 2 cycles of therapy^52^,^53^. Relatedly, the phase II study for eribulin (NCT02097238), another modulator of microtubule dynamics, which is currently enrolling patients, will help determine if the xenograft preclinical system signal translates into some clinical activity. Because MK1775 abrogates the G2/M checkpoint by inhibiting Wee1, there is a clear rationale for a microtubule inhibitor to have increased activity with forced mitotic entry. Interestingly, the MK1775-ixabepilone combination was the only one with substantially different activity when studied in our *ex vivo* assay, perhaps suggesting a protective effect of the microenvironment.

We have attempted at all phases of our methodology to recapitulate human pharmacokinetics in our testing in terms of concentrations, duration, and protein binding. We recognize that for some agents serum levels may over-represent agent delivery to tumor cells and in other situations it may underestimate conditions in the tumor and its microenvironment. Unfortunately, intratumoral concentrations of many agents are not available. We also recognize that not all agent effects can be determined *in vitro* and that we may have dismissed agents with eventual clinical activity. Among tyrosine kinase inhibitors, the most common class of FDA-approved agents and the most common mechanism among our tested agents, we measured little activity. This could be because these agents have anti-angiogenic or stromal effects clinically, which we could not measure *in vitro* or because of clinically targeted kinases not playing a significant role in osteosarcoma tumor cell biology. Sorafenib has promising osteosarcoma activity clinically that is worthy of further exploration but was not very active in our system, for example^54^,^55^. Others have demonstrated better activity of kinase inhibitors *in vivo* than *in vitro* in a neuroblastoma preclinical study, which may be due to effects on angiogenesis or other stromal effects^56^. Nonconjugated immunotherapies or agents with stromal or other non-tumor targets would similarly not be predicted to have any detectable activity in our system as well. Importantly, we acknowledge that this system is intended to explore a number of agents and combinations that could not reasonably be investigated in patients or animal models due to the sheer number of untested agents in osteosarcoma. It is yet to be proven that the methods of incorporating clinically achievable concentrations and modulating levels based on the half-life of agents will be more informative for clinical translation than prior *in vitro* studies that haven’t incorporated human pharmacokinetic data.

The methods presented here demonstrate a comprehensive, reproducible, and high-throughput method for exploring antitumor effects of combinations of therapies. In particular, we discovered that combinations of proteasome and HDAC inhibitors demonstrate excellent activity against osteosarcoma cell lines. The 4 most active agents in these classes are all FDA-approved for other indications, which may help with availability for further study. Additionally, MK1775 with ixabepilone would be interesting for early-phase clinical trials.

Combinations of targeted, targeted and cytotoxic, or multiple cytotoxic agents can be explored with this methodology. Particularly for diseases such as osteosarcoma that have few presently available treatments avenues, this important early preclinical data can serve as the basis for confirmatory assays, explorations into the mechanisms of the most promising agents, canine or xenograft studies, and ultimately clinical trials.

## Methods

### Ethics Statement

All patient samples were obtained in accordance with the guidelines mandated by the Total Cancer Care Protocol as approved by the Institutional Review Board at Moffitt Cancer Center. All patients provided written informed consent prospectively. All procedures involving animals were approved by and carried out in accordance with the guidelines mandated by the Moffitt Cancer Center Institutional Animal Care and Use Committee.

### Investigational agents

Agents used included both cytotoxic and targeted agents (most obtained directly from SelleckChem, Sequoia, and Sigma-Aldrich; Supplemental Table S1). Stock solutions were made for each compound in DMSO at 4000× concentrations used in experiments. Structures for all agents are publicly available.

### Cell culture

Osteosarcoma cell lines were obtained from ATCC (Manassas, VA). Cells were maintained in DMEM with 15% FBS according to manufacturer’s recommendations. Cells were grown at 37 °C and 5% CO_2_. All cell lines tested free of mycoplasma every 3 months with MycoAlert tests (Lonza Rockland, Rockland, ME). Cell line identity was confirmed using StemElite ID system (Promega, Madison, WI) using the manufacturer’s instructions and the ATCC STR profile database.

### Single-agent screening

Single-agent activities of a panel of 54 therapeutic candidates (Supplemental Table S1) were characterized with 5 pediatric osteosarcoma cell lines (143B, MNNG/HOS, MG63, U2OS, Saos2). Human pharmacokinetic data were collected for all agents from previously reported phase I studies, using pediatric and combination studies when available (Supplemental Table S1). For agents with half-lives <8 hours and dosing schedule that was not continuous, agents were applied to the cell lines for 6 hours then removed by 1:125 medium dilution. The remaining agents with longer half-lives were applied for 72 hours. Single-agent anti-tumor activities were assessed at the maximum concentration (C_max_), 20% C_max_, and 4% C_max_ using Caspase-Glo and CellTiter-Glo luminescence assays at 24 and 72 hours.

### Two-drug combination screening

A 5 × 5 checker-board matrix format was used to assess all two-drug combinations at five clinically achievable concentrations. Each combination was evaluated at multiple drug ratios to identify synergy (Supplemental Figure S1A). In cases where the same dilution factors were used for both drugs of the combination, diagonals of the 5 × 5 checker-board matrix provide the effects of the drug combination at constant drug ratio. Full dose-response curves were obtained for each individual drug, and the combination index (CI) for all combinations were calculated using CalcuSyn 2.0 and custom-designed analysis package based on the Chou-Talalay method (Supplemental Figure S1C).

### Cell viability assays

The activity levels of single agents and combinations were determined by a high-throughput CellTiter-Glo cell viability assay (Promega). Cells (1–2 × 10^3^) were plated in each well of 384–well plates using a Precision XS liquid handling station (Bio-Tek Instruments, Winooski, VT) and incubated overnight. Drug source plates were prepared in 96-well Megatiter plates (Neptune Scientific, San Diego, CA), and the Precision XS station was used to transfer drugs to four replicate wells with an additional four control wells receiving DMSO vehicle control without drug. At the end of the drug incubation period, CellTiter-Glo or Caspase-Glo reagent was added to each well at 1:1 ratio (v/v) with media. The luminescence of the product of viable cells was measured with a Synergy 4 microplate reader (Bio-Tek Instruments). The luminescence data were transferred to Microsoft Excel to calculate percent viability. IC50 values were determined using a sigmoidal equilibrium model regression and XLfit version 5.2 (ID Business Solutions). The IC50 values obtained from single-drug cell viability assays were used to design subsequent drug combination experiments. High-throughput two-agent combination screening experiments were performed using a 5 × 5 matrix format in 384-well plates to interrogate 25 individual concentration ratios per combination (Supplemental Figure S1).

### Analysis of additive and synergistic effects in combination screening data

For drug combination experiments, the CellTiter-Glo assay was used to measure cell viability, with results analyzed for synergistic, additive, or antagonistic effects using primarily the CI method of Chou-Talalay^57^ with additional supporting analysis from Fold of Potentiation (FOP) and the MixLow method developed by Boik and colleagues^58^. For the CI method, the dose-effect curve for each drug was determined based on experimental observations using the median-effect principle and was compared to the effect achieved with the 2-drug combination to derive a CI value. This method involves plotting dose-effect curves for each single agent using the median-effect equation: fa/fu = (D/Dm)m, where D = dose of the drug, Dm = dose required for 50% effect, fa and fu = affected and unaffected fractions, respectively (fa = 1−fu), and m = exponent signifying the sigmoidicity of the dose-effect curve. XLfit computer software was used to calculate Dm and m. CIs used for the analysis of the drug combinations were determined by the isobologram equation for mutually nonexclusive drugs that have different modes of action: CI = (D)_1_/(Dx)_1_ + (D)_2_/(Dx)_2_ + (D)_1_(D)_2_/(Dx)_1_(Dx)_2_, where (Dx)_1_ and (Dx)_2_ in the denominators are the doses (or concentrations) for D_1_ (Drug1) and D_2_ (Drug2) alone that gives x% inhibition, whereas (D)_1_ and (D)_2_ in the numerators are the doses of Drug1 and Drug2 in combination that also inhibited x% (i.e., isoeffective). CI calculations were done in custom Microsoft Excel templates and verified with CalcuSyn 2.0 (Biosoft, Cambridge, UK). CI < 1, CI = 1, and CI > 1 indicate synergism, additive effects, and antagonism, respectively.

FOP and Mixlow methods were used to further confirm CI synergy calculations. FOP was used for combination screening data with non-constant molar ratios to demonstrate the enhancement of one drug’s effect by another by measuring shift in IC50^25^,^59^. Curve fitting for FOP was performed using Prism V6.05 (GraphPad Software, La Jolla, CA, www.graphpad.com). Dose-response plots for single agents and drug combinations were fitted using a four-parameter non-linear least-squares regression model. Curves were extrapolated to relevant maximum and minimum response levels. The Mixlow method uses a nonlinear mixed-effects model in combination with simulations of a sham mixture of a drug with itself to provide confidence intervals for the Loewe interaction index. Loewe interaction indices LI < 1, LI = 1, and LI > 1 indicate synergism, additive effects, and antagonism, respectively. Mixlow calculations were done using Mixlow package 1.0^59^.

### Cluster analysis

Prior to clustering, fraction affected (FA) and CI data were log-transformed and normalized to a common scale by multiplying the log-transformed 1-FA by a coefficient of 1/3. To provide values suitable for inputting into subsequent cluster analysis, both variables were multiplied by −10. Cluster analysis was accomplished with the use of Cluster 3.0 (Stanford University Labs, Stanford, CA). Complete-linkage unsupervised hierarchical clustering of FA and CI values together was performed using uncentered absolute correlation similarity metrics. Java TreeView 1.1.6r4 (Stanford University Labs) was employed to visualize clustered data.

### Apoptosis assay

Caspase-3/7 activation was measured using a 384-well plate based Caspase-Glo 3/7 (Promega) luminescent assay. Cells were treated for 24 hours with serial dilutions of each compound and assessed with Caspase-Glo reagent at 1:1 ratio with media. Luminescence was measured with a Synergy 4 microplate reader (Bio-Tek Instruments).

### Tumor xenograft development

Fresh cancer tissues, obtained from surgical specimens of patients undergoing resection for osteosarcoma at the Moffitt Cancer Center, were established as subcutaneous xenografts in female athymic (nu+/nu+) mice (Harlan Laboratories, Washington, DC) (F1 generation). The tumor xenografts from the F1 generation were harvested and reimplanted subcutaneously in groups of five mice for each patient (F2 generation). Tumors were allowed to grow to 1.5 cm, at which point they were harvested, divided into ~3-mm^3^ pieces, and transplanted to another 18–22 mice (F3 generation). Tumors from the F3 generation were grown until reaching ~200 mm^3^ before harvesting for ex vivo assays.

### *Ex vivo* assay

Tumor cells were collected by fine-needle aspiration from the xenograft animals using a sterile 25G short needle. After tumor samples were microscopically confirmed to be enriched for cancer cells, they were immediately transferred into 10 mL sterile prewarmed complete RPMI 1640 culture medium containing 10% FBS, penicillin (200 μg/mL), and streptomycin (200 μg/mL). Cells were stained with trypan blue and suspended in PBS to assess viability. The viable (membrane intact) and dead cells were then counted, and the total viable cell count was used to calculate final working cell concentrations. ~10,000 viable tumor cells were seeded into each well of a 96-well polystyrene microplate. Cells were treated in duplicate with DMSO (vehicle control) or the top six compounds as single agents and 2-drug combinations in a humidified 5% CO_2_ incubator at 37 °C for 72 hours. Cell viability was assessed using CellTiter-Glo.

### Isolation, culture, and osteogenic differentiation of murine mesenchymal stem cells

Isolation and culture of mesenchymal stem cells (MSCs) were adopted from published protocol^60^. Hind legs were collected from 6 non-tumor-bearing 4–6 week-old C57/BL6 Rag2−/− mice and placed in sterile PBS. Excess tissue was removed and bones were rinsed in 75% ethanol and dried. Ends were trimmed and bone marrow was flushed three times with sterile PBS to deplete the hematopoietic cells. Bones were cut into 1–3 mm chips, digested with 1 mg/mL collagenase II (Invitrogen, Carlsbad, CA) in α-MEM with 15% FBS, and shaken 1 hour in 37 °C at 150 RPM. Digested bone fragments were grown in 6-well tissue culture plates in α-MEM with 15% FBS. Medium was changed every 3 days. For osteogenic differentiation, MSCs were seeded at 6,000 cells/well in 48-well plates and cultured until 100% confluent. 20× StemXVivo mouse/rat osteogenic supplement (R&D Systems, #CCM009) was added to media and changed every 2–3 days for 14–21 days Cells were washed with PBS, fixed in 10% buffered formalin for 15 minutes, rinsed twice with ddH_2_O, and then stained for 45 minutes in the dark using 4% alizarin red (Fisher Scientific, #AC400480250) to verify osteogenic differentiation. pH was adjusted to 4.2 using 10% NH_4_OH. After stain was removed, cells were washed 4 times with ddH_2_O and allowed to dry. Bright red stain showing calcium deposits was confirmed visually.

## Additional Information

**How to cite this article**: Yu, D. *et al.* Identification of Synergistic, Clinically Achievable, Combination Therapies for Osteosarcoma. *Sci. Rep.* **5**, 16991; doi: 10.1038/srep16991 (2015).

## Supplementary Material

Supplementary Information

## Figures and Tables

**Table 1:** Select Fa and CI values of top six combinations with order-of-addition.

Select Fa and CI values of top six combinations with order-of-addition.																			
Tx1	Tx2	Conc (ng/ml)		Order of Addition	MNNG/HOS		143B		MG63		U-2 OS		Saos2		FA	*CI*	P-value, N = 10 (FA; CI)		
		Tx1	Tx2		FA	*CI*	FA	*CI*	FA	*CI*	FA	*CI*	FA	*CI*	(mean ± SEM)	(mean ± SEM)	*Con vs*. 1→2	*Con vs*. 2→1	1→2 *vs*. 2→1
Bortezomib	Panobinostat	12	14	*Concurrent*	1.00	*0.43*	1.00	*0.38*	1.00	*0.60*	1.00	*0.12*	0.95	*0.60*	0.99 ± 0.01	*0.44* ± 0.08	0.016; <0.001	0.141; 0.111	0.228; <0.001
				*Tx1 → Tx2*	0.95	*0.85*	1.00	*0.94*	0.99	*0.88*	0.95	*0.42*	0.78	*0.64*	0.94 ± 0.02	*0.76* ± 0.06			
				*Tx2 → Tx1*	0.94	*0.54*	1.00	*0.36*	0.96	*0.44*	1.00	*0.07*	0.99	*0.25*	0.98 ± 0.01	*0.34* ± 0.06			
Bortezomib	Romidepsin	12	200	*Concurrent*	1.00	*0.35*	1.00	*0.91*	1.00	*0.59*	1.00	*0.12*	1.00	*0.48*	1.00 ± 0.00	*0.50* ± 0.09	0.010; 0.001	0.040; 0.010	0.026; <0.001
				*Tx1 → Tx2*	0.97	*0.77*	1.00	*0.82*	0.99	*0.83*	0.98	*0.41*	0.93	*0.99*	0.98 ± 0.01	*0.77* ± 0.07			
				*Tx2 → Tx1*	0.99	*0.36*	1.00	*0.39*	1.00	*0.11*	1.00	*0.07*	1.00	*0.51*	1.00 ± 0.00	*0.27* ± 0.06			
Carfilzomib	Panobinostat	30	14	*Concurrent*	0.98	*0.33*	1.00	*0.36*	1.00	*0.39*	1.00	*0.05*	1.00	*0.23*	1.00 ± 0.00	*0.28* ± 0.07	0.051; <0.001	0.002; 0.004	0.002; 0.090
				*Tx1 → Tx2*	0.91	*0.61*	1.00	*0.58*	1.00	*0.62*	0.99	*0.21*	0.98	*0.54*	0.98 ± 0.01	*0.52* ± 0.08			
				*Tx2 → Tx1*	0.60	*0.78*	0.90	*0.53*	0.81	*1.18*	0.85	*0.75*	1.00	*0.20*	0.83 ± 0.04	*0.73* ± 0.13			
Carfilzomib	Romidepsin	30	200	*Concurrent*	0.99	*0.21*	1.00	*0.49*	1.00	*0.37*	1.00	*0.08*	1.00	*0.60*	1.00 ± 0.00	*0.35* ± 0.07	0.010; 0.003	0.039; 0.459	0.659; 0.022
				*Tx1 → Tx2*	0.93	*0.61*	1.00	*0.65*	1.00	*0.66*	0.96	*0.53*	0.97	*0.65*	0.97 ± 0.01	*0.62* ± 0.08			
				*Tx2 → Tx1*	0.91	*0.55*	1.00	*0.50*	1.00	*0.12*	0.98	*0.34*	1.00	*0.31*	0.98 ± 0.01	*0.34* ± 0.08			
MK1775	Ixabepilone	980	80	*Concurrent*	0.96	*0.85*	0.98	*0.49*	0.87	*1.67*	0.93	*0.50*	0.97	*0.96*	0.94 ± 0.01	*0.89* ± 0.17	0.003; 0.446	0.070; 0.113	0.014; 0.054
				*Tx1 → Tx2*	0.89	*1.29*	0.96	*0.43*	0.82	*1.42*	0.82	*0.99*	0.94	*0.79*	0.88 ± 0.02	*1.02* ± 0.16			
				*Tx2 → Tx1*	0.93	*0.77*	0.97	*0.36*	0.88	*0.75*	0.86	*0.58*	0.96	*0.98*	0.92 ± 0.01	*0.69* ± 0.12			
MK1775	Romidepsin	980	200	*Concurrent*	0.95	*0.80*	0.97	*1.42*	0.94	*0.64*	0.99	*0.16*	0.99	*1.28*	0.97 ± 0.01	*0.86* ± 0.18	0.144; 0.277	0.007; 0.024	0.303; 0.066
				*Tx1 → Tx2*	0.87	*1.46*	0.99	*0.23*	0.92	*0.80*	0.94	*0.65*	0.99	*0.46*	0.94 ± 0.02	*0.73* ± 0.22			
				*Tx2 → Tx1*	0.85	*1.17*	0.79	*3.36*	0.77	*1.53*	0.93	*0.49*	0.99	*1.58*	0.86 ± 0.03	*1.62* ± 0.41			

**Table 2:** *Ex vivo* synergy analysis of top 6 drug combinations at clinically achievable concentrations using xenograft model.

*Ex vivo*synergy analysis of top 6 drug combinations									
		Panobinostat (ng/ml)				Romidepsin (ng/ml)			
		8		20		80		200	
		Fa (%)	*CI*	Fa (%)	*CI*	Fa (%)	*CI*	Fa (%)	*CI*
Bortezomib (ng/ml)	4.8	98.5 ± 0.6	*0.48*	97.8 ± 0.1	*0.68*	96.4 ± 1.9	*0.41*	96.7 ± 0.7	*0.71*
	12	97.9 ± 0.1	*0.38*	96.1 ± 0.1	*0.58*	97.9 ± 0.1	*0.60*	97.4 ± 0.5	*1.43*
Carfilzomib (ng/ml)	12	98.2 ± 0.7	*0.35*	98.5 ± 0.1	*0.79*	98.5 ± 0.2	*0.81*	98.3 ± 0.1	*0.62*
	30	97.4 ± 0.3	*0.26*	97.4 ± 0.1	*0.64*	98.1 ± 0.2	*0.29*	95.2 ± 2.1	*0.52*
		**Ixabepilone (ng/ml)**				**Romidepsin (ng/ml)**			
		**30**		**75**		**80**		**200**	
		Fa (%)	*CI*	Fa (%)	*CI*	Fa (%)	*CI*	Fa (%)	*CI*
MK1775 (ng/ml)	200	31.9 ± 2.7	*0.14*	37.3 ± 6.0	*0.17*	98.9 ± 0.1	*0.29*	98.5 ± 0.3	*0.46*
	500	29.6 ± 5.0	*0.19*	31.4 ± 1.3	*0.32*	98.5 ± 0.1	*0.12*	97.8 ± 0.1	*0.31*
