# Supplementary material for: Identification of Synergistic, Clinically Achievable, Combination Therapies for Osteosarcoma
Source: Sci Rep. 2015 Nov 25;5:16991. doi: 10.1038/srep16991 (PMC4658502; doi:10.1038/srep16991)

Identification of Synergistic, Clinically Achievable, Combination Therapies for Osteosarcoma

Diana Yu[1,2], Elliot Kahen[1,2], Christopher L. Cubitt[2], Jeremy McGuire[4], Jenny Kreahling[4], Jae Lee[4,6], Soner Altiok[4,7], Conor C. Lynch[4,8], Daniel M. Sullivan[2,4,9], and Damon R. Reed[1,3,4,5,*]

[1]Sunshine Lab, H. Lee Moffitt Cancer Center and Research Institute, Tampa, FL 33612 Florida USA

[2]Translational Research Lab, H. Lee Moffitt Cancer Center and Research Institute, Tampa, FL 33612 Florida USA

[3]Sarcoma Department, H. Lee Moffitt Cancer Center and Research Institute, Tampa, FL 33612 Florida USA

[4]Chemical Biology and Molecular Medicine Program, H. Lee Moffitt Cancer Center and Research Institute, Tampa, FL 33612 Florida USA

[5]Adolescent and Young Adult Program, H. Lee Moffitt Cancer Center and Research Institute, Tampa, FL 33612 Florida USA

[6]Department of Biostatistics and Bioinformatics, H. Lee Moffitt Cancer Center and Research Institute, Tampa, FL 33612 Florida USA

[7]Department of Anatomic Pathology, H. Lee Moffitt Cancer Center and Research Institute, Tampa, FL 33612 Florida USA

[8]Department of Tumor Biology, H. Lee Moffitt Cancer Center and Research Institute, Tampa, FL 33612 Florida USA

[9]Department of Blood and Marrow Transplantation, H. Lee Moffitt Cancer Center and Research Institute, Tampa, FL 33612 Florida USA

*Damon.Reed@moffitt.org

Pharmacokinetic Properties of the 54 Chemotherapy Agents Screened Against Osteosarcoma

Chemotherapy Agents	Vendor Source	Mean T1/2 (hrs)	Mean Cmax (ng/ml)	Mean Css (ng/ml)	Top Conc in single-agent screening (ng/ml)	MW (g/mol)	References
**Alkylators**							
Palifosfamide	Sequoia	10	83000	n/a	83000	221.02	Camacho et al., 2009 (1)
Cisplatin	Selleckchem	24	1200	n/a	1200	300.05	Veal et al., 2001 (2)
Temozolomide	Selleckchem	2	10000	n/a	10000	194.15	Horton et al., 2007 (3)
TH-302	Sequoia	1	1000	n/a	1000	449.04	Ganjoo et al., 2011 (4)
Dacarbazine	Selleckchem	5	4000	n/a	4000	182.18	Brendel et al., 2011 (5)
**Antifolates**							
Pralatrexate	Selleckchem	12	10000	10	10	477.47	Krug et al., 2000 (6)
Pemetrexed	Sequoia	3.3	421000	20	421000	427.41	Malempati et al., 2007 (7)
Methotrexate	Sequoia	2	454400	9000	454400	454.44	Zelcer et al., 2008 (8)
**HDAC inhibitors**							
Panobinostat	Selleckchem	29	18.5	5	18.5	349.43	Slingerland et al., 2014 (9)
Romidepsin	Selleckchem	3	377	1	377	540.70	Amiri-Kordestani et al., 2013; Fouladi et al., 2006 (10, 11)
Vorinostat	Selleckchem	5	300	n/a	300	264.32	Muscal et al., 2013 (12)
**Microtubule inhibitors**							
GSK923295A	Sequoia	10	7122	100	7122	592.13	Chung et al., 2012 (13)
Docetaxel	Selleckchem	10	2000	n/a	2000	807.88	Extra et al., 1993 (14)
Ixabepilone	Sequoia	15	100	4.6	100	506.70	Widemann et al., 2009 (15)
Vinorelbine	Selleckchem	15	100	10	20	1079.11	Rahmani et al., 1987 (16)
Eribulin	Sequoia	40	519	n/a	519	826.00	Mukohara et al., 2012 (17)
**TKI's**							
Cabozantinib	Selleckchem	91	2000	n/a	2000	501.51	Kurzrock et al., 2011 (18)
Bosutinib	Selleckchem	24	200	n/a	200	530.45	Rassi & Khoury, 2013 (19)
Ponatinib	Selleckchem	24	73	n/a	73	532.56	Narasimhan et al., 2013 (20)
Lapatinib	Selleckchem	3	5000	1000	5000	925.46	Fouladi et al., 2010 (21)
Crizotinib	Sequoia	42	630	420	630	450.34	Mosse et al., 2013 (22)
Gefitinib	Selleckchem	12	2210	1000	2210	446.90	Daw et al., 2005 (23)
MEK162	Selleckchem	3.6	493	n/a	493	441.23	Finn et al., 2012 (24)
Selumetinib	Selleckchem	6.1	781	n/a	781	457.68	Adjei et al., 2008 (25)
BKM120	Sequoia	24	969	n/a	969	410.39	Bendell et al., 2012 (26)
Vemurafenib	Selleckchem	57	31000	n/a	31000	489.92	Bautista et al., 2014 (27)
Dasatinib	Selleckchem	2.7	146	n/a	146	488.01	Aplenc et al., 2011 (28)
Saracatinib	Selleckchem	39	300	100	300	542.03	Baselga et al., 2010 (29)
Axitinib	Selleckchem	4	27.8	n/a	27.8	386.47	Martin et al., 2012 (30)
Pazopanib	Sequoia	31	15000	7000	15000	473.98	Hurwitz et al., 2009 (31)
Vandetanib	Selleckchem	300	264	748	748	475.35	Broniscer et al., 2010 (32)
Regorafenib	Selleckchem	28	3900	n/a	3900	500.83	Strumberg et al., 2012 (33)
Sorafenib	Sequoia	30	3400	n/a	3400	637.03	Widemann et al., 2012 (34)
Dinaciclib	Sequoia	2	700	10	700	396.49	J. J. Nemunaitis et al., 2013 (35)
MK1775	Sequoia	2	500	n/a	500	500.60	Schellens et al., 2009 (36)
**Topoisomerase inhibitors**							
Topotecan	Sequoia	3	2	1	2	457.91	Daw et al., 2004 (37)
SN-38	Sequoia	11	5	n/a	5	392.40	Furman et al., 2006 (38)
Etoposide	Selleckchem	7	5000	n/a	5000	588.56	Sinkule et al., 1984 (39)
Doxorubicin	Selleckchem	20	40	n/a	40	543.52	Ackland et al., 1989 (40)
**Nucleoside analogues**							
Cytarabine	Selleckchem	2.5	243.2	n/a	243.2	243.22	Avramis et al., 1987 (41)
Gemcitabine	Selleckchem	10	41000	3000	41000	299.66	Reid et al., 2004 (42)
**mTOR inhibitors**							
Everolimus	Selleckchem	30	11	n/a	11	958.22	Fouladi et al., 2007 (43)
Temsirolimus	Sequoia	31	800	100	400	1030.29	Spunt et al., 2011 (44)
BEZ235	Selleckchem	3.5-13.5	2500	1700	47	469.54	Arkenau et al., 2012 (45)
**Other**							
Arsenic Trioxide	Sigma	3	1200	100	200	197.84	Shen et al., 1997 (46)
Pomalidomide	Sequoia	7.5	75	n/a	75	273.24	Li et al., 2015 (47)
Zoledronic acid	Selleckchem	12	167	n/a	167	272.09	Russell et al., 2011 (48)
Plerixafor	Sequoia	4.6	926	200	926	830.50	Stewart et al., 2009 (49)
17-AAG	Selleckchem	3	5300	n/a	5300	585.69	Weigel et al., 2007 (50)
Omacetaxine	Santa Cruz Biotech	6	100	n/a	100	545.62	J. Nemunaitis et al., 2013 (51)
Carfilzomib	Sequoia	1	4232	1	4232	719.91	Papadopoulos et al., 2013 (52)
Bortezomib	Sequoia	12	63	5	63	384.24	Moreau et al., 2012; Muscal et al., 2013 (12, 53)
Vismodegib	Selleckchem	96	4382	8215	8215	421.30	Gajjar et al., 2013 (54)
Azacitidine	Selleckchem	4	750	n/a	750	244.20	van Groeningen et al., 1986 (55)

Supplemental Table S1. Sources and pharmacokinetic properties of the 54 chemotherapy agents screened at clinically achievable concentrations using osteosarcoma cell lines

Supplemental Table S1 References

1. Camacho LH, Chawla SP, Chua V, Abbadessa G, Komarnitsky PB, Lewis J. A phase I study of palifosfamide in combination with doxorubicin: Safety and preliminary efficacy. ASCO Meeting Abstracts. 2009;27(15S):10577.
2. Veal GJ, Dias C, Price L, Parry A, Errington J, Hale J, et al. Influence of cellular factors and pharmacokinetics on the formation of platinum-DNA adducts in leukocytes of children receiving cisplatin therapy. Clin Cancer Res. 2001;7(8):2205-12. Epub 2001/08/08. PubMed PMID: 11489793.
3. Horton TM, Thompson PA, Berg SL, Adamson PC, Ingle AM, Dolan ME, et al. Phase I pharmacokinetic and pharmacodynamic study of temozolomide in pediatric patients with refractory or recurrent leukemia: a Children's Oncology Group Study. J Clin Oncol. 2007;25(31):4922-8. Epub 2007/11/01. doi: 10.1200/jco.2007.12.0667. PubMed PMID: 17971589.
4. Ganjoo KN, Cranmer LD, Butrynski JE, Rushing D, Adkins D, Okuno SH, et al. A phase I study of the safety and pharmacokinetics of the hypoxia-activated prodrug TH-302 in combination with doxorubicin in patients with advanced soft tissue sarcoma. Oncology. 2011;80(1-2):50-6. Epub 2011/06/01. doi: 10.1159/000327739. PubMed PMID: 21625179.
5. Brendel E, Ludwig M, Lathia C, Robert C, Ropert S, Soria JC, et al. Pharmacokinetic results of a phase I trial of sorafenib in combination with dacarbazine in patients with advanced solid tumors. Cancer Chemother Pharmacol. 2011;68(1):53-61. doi: 10.1007/s00280-010-1423-9. PubMed PMID: 20821331; PubMed Central PMCID: PMC3123694.
6. Krug LM, Ng KK, Kris MG, Miller VA, Tong W, Heelan RT, et al. Phase I and pharmacokinetic study of 10-propargyl-10-deazaaminopterin, a new antifolate. Clin Cancer Res. 2000;6(9):3493-8. Epub 2000/09/22. PubMed PMID: 10999734.
7. Malempati S, Nicholson HS, Reid JM, Blaney SM, Ingle AM, Krailo M, et al. Phase I trial and pharmacokinetic study of pemetrexed in children with refractory solid tumors: the Children's Oncology Group. J Clin Oncol. 2007;25(12):1505-11. Epub 2007/04/20. doi: 10.1200/jco.2006.09.1694. PubMed PMID: 17442992.
8. Zelcer S, Kellick M, Wexler LH, Gorlick R, Meyers PA. The Memorial Sloan Kettering Cancer Center experience with outpatient administration of high dose methotrexate with leucovorin rescue. Pediatr Blood Cancer. 2008;50(6):1176-80. Epub 2008/02/13. doi: 10.1002/pbc.21419. PubMed PMID: 18266225.
9. Slingerland M, Hess D, Clive S, Sharma S, Sandstrom P, Loman N, et al. A phase I, open-label, multicenter study to evaluate the pharmacokinetics and safety of oral panobinostat in patients with advanced solid tumors and various degrees of hepatic function. Cancer Chemother Pharmacol. 2014;74(5):1089-98. Epub 2014/09/26. doi: 10.1007/s00280-014-2594-6. PubMed PMID: 25253045.
10. Fouladi M, Furman WL, Chin T, Freeman BB, 3rd, Dudkin L, Stewart CF, et al. Phase I study of depsipeptide in pediatric patients with refractory solid tumors: a Children's Oncology Group report. J Clin Oncol. 2006;24(22):3678-85. Epub 2006/08/01. doi: 10.1200/jco.2006.06.4964. PubMed PMID: 16877737.
11. Amiri-Kordestani L, Luchenko V, Peer CJ, Ghafourian K, Reynolds J, Draper D, et al. Phase I trial of a new schedule of romidepsin in patients with advanced cancers. Clin Cancer Res. 2013;19(16):4499-507. Epub 2013/06/13. doi: 10.1158/1078-0432.ccr-13-0095. PubMed PMID: 23757352; PubMed Central PMCID: PMCPMC3967244.
12. Muscal JA, Thompson PA, Horton TM, Ingle AM, Ahern CH, McGovern RM, et al. A phase I trial of vorinostat and bortezomib in children with refractory or recurrent solid tumors: a Children's Oncology Group phase I consortium study (ADVL0916). Pediatr Blood Cancer. 2013;60(3):390-5. Epub 2012/08/14. doi: 10.1002/pbc.24271. PubMed PMID: 22887890; PubMed Central PMCID: PMCPMC3511610.
13. Chung V, Heath EI, Schelman WR, Johnson BM, Kirby LC, Lynch KM, et al. First-time-in-human study of GSK923295, a novel antimitotic inhibitor of centromere-associated protein E (CENP-E), in patients with refractory cancer. Cancer Chemother Pharmacol. 2012;69(3):733-41. Epub 2011/10/25. doi: 10.1007/s00280-011-1756-z. PubMed PMID: 22020315.
14. Extra JM, Rousseau F, Bruno R, Clavel M, Le Bail N, Marty M. Phase I and pharmacokinetic study of Taxotere (RP 56976; NSC 628503) given as a short intravenous infusion. Cancer Res. 1993;53(5):1037-42. Epub 1993/03/01. PubMed PMID: 8094996.
15. Widemann BC, Goodspeed W, Goodwin A, Fojo T, Balis FM, Fox E. Phase I trial and pharmacokinetic study of ixabepilone administered daily for 5 days in children and adolescents with refractory solid tumors. J Clin Oncol. 2009;27(4):550-6. Epub 2008/12/17. doi: 10.1200/jco.2008.17.6644. PubMed PMID: 19075272; PubMed Central PMCID: PMCPMC2645861.
16. Rahmani R, Bruno R, Iliadis A, Favre R, Just S, Barbet J, et al. Clinical pharmacokinetics of the antitumor drug navelbine (5'-noranhydrovinblastine). Cancer Res. 1987;47(21):5796-9. Epub 1987/11/01. PubMed PMID: 3664483.
17. Mukohara T, Nagai S, Mukai H, Namiki M, Minami H. Eribulin mesylate in patients with refractory cancers: a Phase I study. Invest New Drugs. 2012;30(5):1926-33. Epub 2011/09/03. doi: 10.1007/s10637-011-9741-2. PubMed PMID: 21887501; PubMed Central PMCID: PMCPMC3432792.
18. Kurzrock R, Sherman SI, Ball DW, Forastiere AA, Cohen RB, Mehra R, et al. Activity of XL184 (Cabozantinib), an oral tyrosine kinase inhibitor, in patients with medullary thyroid cancer. J Clin Oncol. 2011;29(19):2660-6. Epub 2011/05/25. doi: 10.1200/jco.2010.32.4145. PubMed PMID: 21606412; PubMed Central PMCID: PMCPMC3646303.
19. Rassi FE, Khoury HJ. Bosutinib: a SRC-ABL tyrosine kinase inhibitor for treatment of chronic myeloid leukemia. Pharmgenomics Pers Med. 2013;6:57-62. Epub 2013/09/11. doi: 10.2147/pgpm.s32145. PubMed PMID: 24019749; PubMed Central PMCID: PMCPMC3760444.
20. Narasimhan NI, Dorer DJ, Niland K, Haluska F, Sonnichsen D. Effects of food on the pharmacokinetics of ponatinib in healthy subjects. J Clin Pharm Ther. 2013;38(6):440-4. Epub 2013/07/31. doi: 10.1111/jcpt.12082. PubMed PMID: 23888935; PubMed Central PMCID: PMCPMC4286001.
21. Fouladi M, Stewart CF, Blaney SM, Onar-Thomas A, Schaiquevich P, Packer RJ, et al. Phase I trial of lapatinib in children with refractory CNS malignancies: a Pediatric Brain Tumor Consortium study. J Clin Oncol. 2010;28(27):4221-7. Epub 2010/08/18. doi: 10.1200/jco.2010.28.4687. PubMed PMID: 20713864; PubMed Central PMCID: PMCPMC2953974.
22. Mosse YP, Lim MS, Voss SD, Wilner K, Ruffner K, Laliberte J, et al. Safety and activity of crizotinib for paediatric patients with refractory solid tumours or anaplastic large-cell lymphoma: a Children's Oncology Group phase 1 consortium study. Lancet Oncol.

2013;14(6):472-80. Epub 2013/04/20. doi: 10.1016/s1470-2045(13)70095-0. PubMed PMID: 23598171; PubMed Central PMCID: PMCPMC3730818.
23. Daw NC, Furman WL, Stewart CF, Iacono LC, Krailo M, Bernstein ML, et al. Phase I and pharmacokinetic study of gefitinib in children with refractory solid tumors: a Children's Oncology Group Study. J Clin Oncol. 2005;23(25):6172-80. Epub 2005/09/02. doi: 10.1200/jco.2005.11.429. PubMed PMID: 16135484.
24. Finn RS, Javle MM, Tan BR, Weekes CD, Bendell JC, Patnaik A, et al. A phase I study of MEK inhibitor MEK162 (ARRY-438162) in patients with biliary tract cancer. ASCO Meeting Abstracts. 2012;30(4_suppl):220.
25. Adjei AA, Cohen RB, Franklin W, Morris C, Wilson D, Molina JR, et al. Phase I pharmacokinetic and pharmacodynamic study of the oral, small-molecule mitogen-activated protein kinase kinase 1/2 inhibitor AZD6244 (ARRY-142886) in patients with advanced cancers. J Clin Oncol. 2008;26(13):2139-46. Epub 2008/04/09. doi: 10.1200/jco.2007.14.4956. PubMed PMID: 18390968; PubMed Central PMCID: PMCPMC2718422.
26. Bendell JC, Rodon J, Burris HA, de Jonge M, Verweij J, Birle D, et al. Phase I, dose-escalation study of BKM120, an oral pan-Class I PI3K inhibitor, in patients with advanced solid tumors. J Clin Oncol. 2012;30(3):282-90. Epub 2011/12/14. doi: 10.1200/jco.2011.36.1360. PubMed PMID: 22162589.
27. Bautista F, Paci A, Minard-Colin V, Dufour C, Grill J, Lacroix L, et al. Vemurafenib in pediatric patients with BRAFV600E mutated high-grade gliomas. Pediatr Blood Cancer. 2014;61(6):1101-3. Epub 2014/01/01. doi: 10.1002/pbc.24891. PubMed PMID: 24375920.
28. Aplenc R, Blaney SM, Strauss LC, Balis FM, Shusterman S, Ingle AM, et al. Pediatric phase I trial and pharmacokinetic study of dasatinib: a report from the children's oncology group phase I consortium. J Clin Oncol. 2011;29(7):839-44. Epub 2011/01/26. doi: 10.1200/jco.2010.30.7231. PubMed PMID: 21263099; PubMed Central PMCID: PMCPMC3068059.
29. Baselga J, Cervantes A, Martinelli E, Chirivella I, Hoekman K, Hurwitz HI, et al. Phase I safety, pharmacokinetics, and inhibition of SRC activity study of saracatinib in patients with solid tumors. Clin Cancer Res. 2010;16(19):4876-83. Epub 2010/09/02. doi: 10.1158/1078-0432.ccr-10-0748. PubMed PMID: 20805299.
30. Martin LP, Kozloff MF, Herbst RS, Samuel TA, Kim S, Rosbrook B, et al. Phase I study of axitinib combined with paclitaxel, docetaxel or capecitabine in patients with advanced solid tumours. Br J Cancer. 2012;107(8):1268-76. Epub 2012/09/22. doi: 10.1038/bjc.2012.407. PubMed PMID: 22996612; PubMed Central PMCID: PMCPMC3494424.
31. Hurwitz HI, Dowlati A, Saini S, Savage S, Suttle AB, Gibson DM, et al. Phase I trial of pazopanib in patients with advanced cancer. Clin Cancer Res. 2009;15(12):4220-7. Epub 2009/06/11. doi: 10.1158/1078-0432.ccr-08-2740. PubMed PMID: 19509175.
32. Broniscer A, Baker JN, Tagen M, Onar-Thomas A, Gilbertson RJ, Davidoff AM, et al. Phase I study of vandetanib during and after radiotherapy in children with diffuse intrinsic pontine glioma. J Clin Oncol. 2010;28(31):4762-8. Epub 2010/10/06. doi: 10.1200/jco.2010.30.3545. PubMed PMID: 20921456; PubMed Central PMCID: PMCPMC3020706.
33. Strumberg D, Scheulen ME, Schultheis B, Richly H, Frost A, Buchert M, et al. Regorafenib (BAY 73-4506) in advanced colorectal cancer: a phase I study. Br J Cancer. 2012;106(11):1722-7. Epub 2012/05/10. doi: 10.1038/bjc.2012.153. PubMed PMID: 22568966; PubMed Central PMCID: PMCPMC3364125.
34. Widemann BC, Kim A, Fox E, Baruchel S, Adamson PC, Ingle AM, et al. A phase I trial and pharmacokinetic study of sorafenib in children with refractory solid tumors or leukemias: a Children's Oncology Group Phase I Consortium report. Clin Cancer Res. 2012;18(21):6011-22. Epub 2012/09/11. doi: 10.1158/1078-0432.ccr-11-3284. PubMed PMID: 22962440; PubMed Central PMCID: PMCPMC4008314.
35. Nemunaitis JJ, Small KA, Kirschmeier P, Zhang D, Zhu Y, Jou YM, et al. A first-in-human, phase 1, dose-escalation study of dinaciclib, a novel cyclin-dependent kinase inhibitor, administered weekly in subjects with advanced malignancies. J Transl Med. 2013;11:259. Epub 2013/10/18. doi: 10.1186/1479-5876-11-259. PubMed PMID: 24131779; PubMed Central PMCID: PMCPMC3853718.
36. Schellens JH, Leijen S, Shapiro GI, Pavlick AC, Tibes R, O'Day SJ, et al. A phase I and pharmacological study of MK-1775, a Wee1 tyrosine kinase inhibitor, in both monotherapy and in combination with gemcitabine, cisplatin, or carboplatin in patients with advanced solid tumors. ASCO Meeting Abstracts. 2009;27(15S):3510.
37. Daw NC, Santana VM, Iacono LC, Furman WL, Hawkins DR, Houghton PJ, et al. Phase I and pharmacokinetic study of topotecan administered orally once daily for 5 days for 2 consecutive weeks to pediatric patients with refractory solid tumors. J Clin Oncol. 2004;22(5):829-37. Epub 2004/03/03. doi: 10.1200/jco.2004.07.110. PubMed PMID: 14990638.
38. Furman WL, Crews KR, Billups C, Wu J, Gajjar AJ, Daw NC, et al. Cefixime allows greater dose escalation of oral irinotecan: a phase I study in pediatric patients with refractory solid tumors. J Clin Oncol. 2006;24(4):563-70. Epub 2006/02/01. doi: 10.1200/jco.2005.03.2847. PubMed PMID: 16446328.
39. Sinkule JA, Hutson P, Hayes FA, Etcubanas E, Evans W. Pharmacokinetics of etoposide (VP16) in children and adolescents with refractory solid tumors. Cancer Res. 1984;44(7):3109-13. Epub 1984/07/01. PubMed PMID: 6539169.
40. Ackland SP, Ratain MJ, Vogelzang NJ, Choi KE, Ruane M, Sinkule JA. Pharmacokinetics and pharmacodynamics of long-term continuous-infusion doxorubicin. Clin Pharmacol Ther. 1989;45(4):340-7. Epub 1989/04/01. PubMed PMID: 2702792.
41. Avramis VI, Biener R, Krailo M, Finklestein J, Ettinger L, Willoughby M, et al. Biochemical pharmacology of high dose 1-beta-D-arabinofuranosylcytosine in childhood acute leukemia. Cancer Res. 1987;47(24 Pt 1):6786-92. Epub 1987/12/15. PubMed PMID: 3479250.
42. Reid JM, Qu W, Safgren SL, Ames MM, Krailo MD, Seibel NL, et al. Phase I trial and pharmacokinetics of gemcitabine in children with advanced solid tumors. J Clin Oncol. 2004;22(12):2445-51. Epub 2004/06/16. doi: 10.1200/jco.2004.10.142. PubMed PMID: 15197207.
43. Fouladi M, Laningham F, Wu J, O'Shaughnessy MA, Molina K, Broniscer A, et al. Phase I study of everolimus in pediatric patients with refractory solid tumors. J Clin Oncol. 2007;25(30):4806-12. Epub 2007/10/20. doi: 10.1200/jco.2007.11.4017. PubMed PMID: 17947729.
44. Spunt SL, Grupp SA, Vik TA, Santana VM, Greenblatt DJ, Clancy J, et al. Phase I study of temsirolimus in pediatric patients with recurrent/refractory solid tumors. J Clin Oncol. 2011;29(21):2933-40. Epub 2011/06/22. doi: 10.1200/jco.2010.33.4649. PubMed PMID: 21690471; PubMed Central PMCID: PMCPMC3138720.

45. Arkenau H-T, Jones SF, Kurkjian C, Infante JR, Pant S, Burris HA, et al. The PI3K/mTOR inhibitor BEZ235 given twice daily for the treatment of patients (pts) with advanced solid tumors. ASCO Meeting Abstracts. 2012;30(15_suppl):3097.
46. Shen ZX, Chen GQ, Ni JH, Li XS, Xiong SM, Qiu QY, et al. Use of arsenic trioxide (As2O3) in the treatment of acute promyelocytic leukemia (APL): II. Clinical efficacy and pharmacokinetics in relapsed patients. Blood. 1997;89(9):3354-60. Epub 1997/05/01. PubMed PMID: 9129042.
47. Li Y, Xu Y, Liu L, Wang X, Palmisano M, Zhou S. Population pharmacokinetics of pomalidomide. J Clin Pharmacol. 2015. Epub 2015/01/06. doi: 10.1002/jcph.455. PubMed PMID: 25556560.
48. Russell HV, Groshen SG, Ara T, DeClerck YA, Hawkins R, Jackson HA, et al. A phase I study of zoledronic acid and low-dose cyclophosphamide in recurrent/refractory neuroblastoma: a new approaches to neuroblastoma therapy (NANT) study. Pediatr Blood Cancer. 2011;57(2):275-82. doi: 10.1002/pbc.22821. PubMed PMID: 21671363; PubMed Central PMCID: PMC3117015.
49. Stewart DA, Smith C, MacFarland R, Calandra G. Pharmacokinetics and pharmacodynamics of plerixafor in patients with non-Hodgkin lymphoma and multiple myeloma. Biol Blood Marrow Transplant. 2009;15(1):39-46. Epub 2009/01/13. doi: 10.1016/j.bbmt.2008.10.018. PubMed PMID: 19135941.
50. Weigel BJ, Blaney SM, Reid JM, Safgren SL, Bagatell R, Kersey J, et al. A phase I study of 17-allylaminogeldanamycin in relapsed/refractory pediatric patients with solid tumors: a Children's Oncology Group study. Clin Cancer Res. 2007;13(6):1789-93. Epub 2007/03/17. doi: 10.1158/1078-0432.ccr-06-2270. PubMed PMID: 17363534.
51. Nemunaitis J, Mita A, Stephenson J, Mita MM, Sarantopoulos J, Padmanabhan-Iyer S, et al. Pharmacokinetic study of omacetaxine mepesuccinate administered subcutaneously to patients with advanced solid and hematologic tumors. Cancer Chemother Pharmacol. 2013;71(1):35-41. Epub 2012/10/12. doi: 10.1007/s00280-012-1963-2. PubMed PMID: 23053254; PubMed Central PMCID: PMCPMC3535355.
52. Papadopoulos KP, Burris HA, 3rd, Gordon M, Lee P, Sausville EA, Rosen PJ, et al. A phase I/II study of carfilzomib 2-10-min infusion in patients with advanced solid tumors. Cancer Chemother Pharmacol. 2013;72(4):861-8. Epub 2013/08/27. doi: 10.1007/s00280-013-2267-x. PubMed PMID: 23975329; PubMed Central PMCID: PMCPMC3784064.
53. Moreau P, Karamanesht, II, Domnikova N, Kyselyova MY, Vilchevska KV, Doronin VA, et al. Pharmacokinetic, pharmacodynamic and covariate analysis of subcutaneous versus intravenous administration of bortezomib in patients with relapsed multiple myeloma. Clin Pharmacokinet. 2012;51(12):823-9. Epub 2012/09/29. doi: 10.1007/s40262-012-0010-0. PubMed PMID: 23018466.
54. Gajjar A, Stewart CF, Ellison DW, Kaste S, Kun LE, Packer RJ, et al. Phase I study of vismodegib in children with recurrent or refractory medulloblastoma: a pediatric brain tumor consortium study. Clin Cancer Res. 2013;19(22):6305-12. Epub 2013/10/01. doi: 10.1158/1078-0432.ccr-13-1425. PubMed PMID: 24077351; PubMed Central PMCID: PMCPMC3856244.
55. van Groeningen CJ, Leyva A, O'Brien AM, Gall HE, Pinedo HM. Phase I and pharmacokinetic study of 5-aza-2'-deoxycytidine (NSC 127716) in cancer patients. Cancer Res. 1986;46(9):4831-6. Epub 1986/09/01. PubMed PMID: 2425959.

Combinations that Produce FA Values of At Least 0.85 in All 5 Osteosarcoma Cell Lines																	
Agent 1		Agent 2		Conc Index		U2OS		SAOS2		143B		MNNG		MG63		Average	
Tx1	Mechanism	Tx2	Mechanism	Tx1	Tx2	Fa	CI	Fa	CI	Fa	CI	Fa	CI	Fa	CI	Fa	CI
**Bortezomib**	*Proteasome*	**Carfilzomib**	*Proteasome*	3	5	**0.966**	*0.659*	**0.955**	*0.732*	**0.999**	*1.020*	**0.986**	*0.559*	**0.997**	*0.484*	**0.981**	*0.691*
				4	5	**0.975**	*0.700*	**0.975**	*0.609*	**0.999**	*1.210*	**0.992**	*0.551*	**0.997**	*0.554*	**0.988**	*0.725*
				5	4	**0.959**	*0.824*	**0.904**	*0.696*	**0.999**	*1.110*	**0.989**	*0.606*	**0.991**	*0.737*	**0.969**	*0.795*
				5	5	**0.988**	*0.666*	**0.989**	*0.461*	**0.999**	*1.480*	**0.996**	*0.550*	**0.997**	*0.669*	**0.994**	*0.765*
		**GSK923295A**	*Cell Cycle*	5	1	**0.863**	*0.942*	**0.854**	*2.240*	**0.968**	*1.580*	**0.947**	*0.927*	**0.980**	*0.692*	**0.922**	*1.276*
		**Ixabepilone**	*Microtubules*	5	4	**0.868**	*1.240*	**0.870**	*0.520*	**0.983**	*1.430*	**0.975**	*0.601*	**0.937**	*0.952*	**0.927**	*0.949*
				5	5	**0.853**	*1.710*	**0.870**	*0.693*	**0.986**	*1.390*	**0.977**	*0.595*	**0.943**	*0.935*	**0.926**	*1.065*
		**MK1775**	*Cell Cycle*	3	5	**0.929**	*0.763*	**0.914**	*0.851*	**0.997**	*1.060*	**0.980**	*0.765*	**0.880**	*1.010*	**0.940**	*0.890*
				4	5	**0.968**	*0.638*	**0.922**	*0.831*	**0.998**	*1.110*	**0.988**	*0.731*	**0.935**	*0.898*	**0.962**	*0.842*
				5	5	**0.985**	*0.594*	**0.923**	*0.890*	**0.999**	*1.330*	**0.995**	*0.645*	**0.981**	*0.709*	**0.977**	*0.834*
		**Panobinostat**	*HDAC*	4	5	**1.000**	*0.100*	**0.942**	*0.441*	**1.000**	*0.472*	**0.984**	*0.391*	**0.998**	*0.267*	**0.985**	*0.334*
				5	3	**0.999**	*0.205*	**0.910**	*0.371*	**1.000**	*0.663*	**0.981**	*0.551*	**0.995**	*0.446*	**0.977**	*0.447*
				5	4	**1.000**	*0.178*	**0.964**	*0.246*	**1.000**	*0.437*	**0.991**	*0.452*	**0.997**	*0.381*	**0.991**	*0.339*
				5	5	**0.999**	*0.226*	**0.991**	*0.132*	**1.000**	*0.551*	**0.997**	*0.363*	**0.999**	*0.309*	**0.997**	*0.316*
		**Regorafenib**	*RTK/GF*	5	5	**0.999**	*0.329*	**0.938**	*0.920*	**1.000**	*0.772*	**0.989**	*0.670*	**0.920**	*1.260*	**0.969**	*0.790*
		**Romidepsin**	*HDAC*	1	5	**0.997**	*0.109*	**0.894**	*0.890*	**0.998**	*0.316*	**0.871**	*0.594*	**0.962**	*0.434*	**0.944**	*0.469*
				2	5	**0.999**	*0.096*	**0.898**	*0.899*	**1.000**	*0.301*	**0.953**	*0.362*	**0.996**	*0.185*	**0.969**	*0.369*
				3	5	**0.999**	*0.121*	**0.935**	*0.662*	**1.000**	*0.357*	**0.990**	*0.267*	**0.998**	*0.203*	**0.984**	*0.322*
				4	5	**0.999**	*0.145*	**0.984**	*0.257*	**1.000**	*0.455*	**0.998**	*0.248*	**0.998**	*0.247*	**0.996**	*0.270*
				5	4	**0.999**	*0.225*	**0.935**	*0.486*	**1.000**	*0.718*	**0.999**	*0.278*	**0.998**	*0.348*	**0.986**	*0.411*
				5	5	**1.000**	*0.178*	**0.997**	*0.093*	**1.000**	*0.650*	**0.999**	*0.268*	**0.998**	*0.319*	**0.999**	*0.302*
**Carfilzomib**	*Proteasome*	**Panobinostat**	*HDAC*	5	3	**0.920**	*0.497*	**0.996**	*0.284*	**0.999**	*0.539*	**0.896**	*0.707*	**0.989**	*0.465*	**0.960**	*0.498*
				5	4	**0.987**	*0.217*	**1.000**	*0.114*	**0.999**	*0.452*	**0.946**	*0.517*	**0.997**	*0.273*	**0.986**	*0.315*
				5	5	**0.998**	*0.088*	**1.000**	*0.140*	**1.000**	*0.269*	**0.971**	*0.394*	**0.999**	*0.209*	**0.993**	*0.220*
		**Regorafenib**	*RTK/GF*	5	4	**0.992**	*0.356*	**0.879**	*1.430*	**0.999**	*0.606*	**0.956**	*0.670*	**0.994**	*0.417*	**0.964**	*0.696*
				5	5	**1.000**	*0.146*	**0.996**	*0.424*	**1.000**	*0.408*	**0.990**	*0.433*	**0.892**	*1.460*	**0.976**	*0.574*
		**Romidepsin**	*HDAC*	4	5	**0.994**	*0.103*	**0.957**	*0.716*	**1.000**	*0.158*	**0.954**	*0.319*	**0.996**	*0.167*	**0.980**	*0.293*
				5	2	**0.906**	*0.445*	**0.936**	*3.640*	**0.988**	*0.731*	**0.943**	*0.100*	**0.929**	*0.165*	**0.941**	*1.016*
				5	4	**0.993**	*0.179*	**0.984**	*0.551*	**1.000**	*0.333*	**0.994**	*0.196*	**0.998**	*0.228*	**0.994**	*0.297*
				5	5	**0.999**	*0.062*	**0.999**	*0.237*	**1.000**	*0.259*	**0.995**	*0.184*	**0.999**	*0.212*	**0.998**	*0.191*
**MK1775**	*Cell Cycle (Wee1 Inhibitor)*	**Carfilzomib**	*Proteasome*	5	4	**0.872**	*0.836*	**0.926**	*1.010*	**0.997**	*0.803*	**0.983**	*0.579*	**0.943**	*0.619*	**0.944**	*0.769*
				5	5	**0.948**	*0.657*	**0.939**	*1.240*	**0.999**	*0.712*	**0.992**	*0.533*	**0.992**	*0.429*	**0.974**	*0.714*
		**Gemcitabine**	*Nucleoside*	4	5	**0.892**	*0.238*	**0.873**	*0.983*	**0.968**	*5.730*	**0.883**	*0.577*	**0.873**	*0.226*	**0.898**	*1.551*
				5	4	**0.911**	*0.408*	**0.926**	*0.691*	**0.980**	*2.980*	**0.902**	*1.040*	**0.874**	*0.411*	**0.919**	*1.106*
				5	5	**0.930**	*0.337*	**0.916**	*0.881*	**0.978**	*5.320*	**0.918**	*0.943*	**0.901**	*0.321*	**0.928**	*1.560*
		**Ixabepilone**	*Microtubules*	4	4	**0.864**	*0.614*	**0.938**	*0.334*	**0.960**	*0.554*	**0.931**	*0.479*	**0.876**	*0.274*	**0.914**	*0.451*
				4	5	**0.871**	*0.872*	**0.940**	*0.387*	**0.969**	*0.510*	**0.928**	*0.545*	**0.880**	*0.336*	**0.918**	*0.530*
				5	1	**0.851**	*0.666*	**0.918**	*0.696*	**0.976**	*0.914*	**0.953**	*0.715*	**0.860**	*0.460*	**0.911**	*0.690*
				5	2	**0.870**	*0.625*	**0.927**	*0.642*	**0.975**	*0.928*	**0.956**	*0.694*	**0.867**	*0.447*	**0.919**	*0.667*
				5	3	**0.897**	*0.562*	**0.960**	*0.398*	**0.976**	*0.910*	**0.953**	*0.727*	**0.880**	*0.417*	**0.933**	*0.603*
				5	4	**0.898**	*0.655*	**0.961**	*0.412*	**0.983**	*0.811*	**0.952**	*0.746*	**0.888**	*0.412*	**0.936**	*0.607*
				5	5	**0.895**	*0.887*	**0.954**	*0.515*	**0.986**	*0.769*	**0.952**	*0.778*	**0.898**	*0.420*	**0.937**	*0.674*
		**Panobinostat**	*HDAC*	5	1	**0.869**	*0.563*	**0.946**	*0.500*	**0.961**	*1.070*	**0.938**	*0.818*	**0.899**	*0.317*	**0.923**	*0.654*
				5	2	**0.894**	*0.486*	**0.945**	*0.526*	**0.958**	*1.110*	**0.930**	*0.868*	**0.902**	*0.310*	**0.926**	*0.660*
				5	3	**0.920**	*0.401*	**0.952**	*0.503*	**0.940**	*1.260*	**0.910**	*0.987*	**0.904**	*0.310*	**0.925**	*0.692*
				5	4	**0.945**	*0.313*	**0.931**	*0.757*	**0.910**	*1.470*	**0.866**	*1.230*	**0.897**	*0.347*	**0.910**	*0.823*
		**Regorafenib**	*RTK/GF*	5	5	**0.985**	*0.593*	**0.928**	*1.390*	**0.996**	*0.735*	**0.927**	*1.410*	**0.892**	*0.634*	**0.946**	*0.952*
		**Romidepsin**	*HDAC*	4	4	**0.971**	*0.160*	**0.867**	*1.000*	**0.926**	*0.811*	**0.893**	*0.663*	**0.889**	*0.593*	**0.909**	*0.645*
				4	5	**0.980**	*0.158*	**0.948**	*0.697*	**0.945**	*0.819*	**0.902**	*0.735*	**0.922**	*0.630*	**0.939**	*0.608*
				5	1	**0.925**	*0.382*	**0.915**	*0.743*	**0.976**	*0.925*	**0.958**	*0.675*	**0.866**	*0.494*	**0.928**	*0.644*
				5	2	**0.949**	*0.300*	**0.920**	*0.746*	**0.974**	*0.950*	**0.947**	*0.762*	**0.862**	*0.583*	**0.931**	*0.668*
				5	3	**0.965**	*0.244*	**0.928**	*0.756*	**0.966**	*1.070*	**0.945**	*0.794*	**0.875**	*0.646*	**0.936**	*0.702*
				5	4	**0.973**	*0.235*	**0.955**	*0.627*	**0.971**	*1.040*	**0.941**	*0.851*	**0.888**	*0.775*	**0.945**	*0.706*
				5	5	**0.983**	*0.200*	**0.976**	*0.508*	**0.973**	*1.070*	**0.936**	*0.953*	**0.930**	*0.651*	**0.960**	*0.676*
**Romidepsin**	*HDAC*	**Gemcitabine**	*Nucleoside*	4	4	**0.852**	*0.420*	**0.921**	*2.410*	**0.993**	*2.360*	**0.919**	*0.087*	**0.931**	*0.087*	**0.923**	*1.073*
				4	5	**0.854**	*0.413*	**0.930**	*2.340*	**0.994**	*4.330*	**0.919**	*0.095*	**0.895**	*0.215*	**0.918**	*1.479*
				5	1	**0.879**	*0.631*	**0.923**	*4.250*	**0.974**	*0.556*	**0.938**	*0.111*	**0.901**	*0.302*	**0.923**	*1.170*
				5	3	**0.919**	*0.369*	**0.950**	*3.000*	**0.993**	*1.160*	**0.942**	*0.104*	**0.933**	*0.152*	**0.947**	*0.957*
				5	4	**0.929**	*0.309*	**0.957**	*2.690*	**0.994**	*2.150*	**0.937**	*0.118*	**0.938**	*0.137*	**0.951**	*1.081*
				5	5	**0.926**	*0.324*	**0.967**	*2.210*	**0.992**	*4.770*	**0.939**	*0.118*	**0.909**	*0.287*	**0.947**	*1.542*
		**GSK923295A**	*Cell Cycle*	5	3	**0.917**	*0.379*	**0.972**	*2.210*	**0.879**	*0.427*	**0.851**	*2.260*	**0.851**	*16.400*	**0.894**	*4.335*
				5	4	**0.934**	*0.280*	**0.969**	*2.810*	**0.893**	*0.381*	**0.852**	*4.120*	**0.860**	*28.000*	**0.902**	*7.118*
				5	5	**0.949**	*0.203*	**0.966**	*3.950*	**0.906**	*0.341*	**0.856**	*7.540*	**0.899**	*27.200*	**0.915**	*7.847*

Supplemental Table S2. FA and CI values of drug combinations tested across 5 osteosarcoma cell lines with FA>0.85. Tx1 and Tx2 indices indicate drug concentrations used in the combination as summarized in Supplemental Table S3.

Concentrations of Agents in Combination that Produced FA Values of At Least 0.85 in All Five Osteosarcoma Cell Lines													
Agent Name		Concentration Index		Concentration of Agent Used in a Given Cell Line (ng/ml)									
				U2OS		SAOS2		143B		MNNG		MG63	
Tx1	Tx2	Tx1	Tx2	Tx1	Tx2	Tx1	Tx2	Tx1	Tx2	Tx1	Tx2	Tx1	Tx2
Bortezomib	Carfilzomib	3	5	6.59	30	6.59	30	6.59	30	6.59	30	2.74	30
		4	5	8.89	30	8.89	30	8.89	30	8.89	30	3.7	30
		5	4	12	15	12	15	12	15	12	15	5	15
		5	5	12	30	12	30	12	30	12	30	5	30
	GSK923295A	5	1	12	62.5	12	438	12	438	12	438	5	438
	Ixabepilone	5	4	12	50	12	10	12	10	12	50	5	12.5
		5	5	12	100	12	20	12	20	12	100	5	25
	MK1775	3	5	6.59	980	6.59	980	6.59	980	6.59	980	2.74	980
		4	5	8.89	980	8.89	980	8.89	980	8.89	980	3.7	980
		5	5	12	980	12	980	12	980	12	980	5	980
	Panobinostat	4	5	8.89	14	8.89	14	8.89	14	8.89	14	3.7	14
		5	3	12	3.5	12	3.5	12	3.5	12	3.5	5	3.5
		5	4	12	7	12	7	12	7	12	7	5	7
		5	5	12	14	12	14	12	14	12	14	5	14
	Regorafenib	5	5	12	15000	12	15000	12	15000	12	15000	5	3900
	Romidepsin	1	5	3.61	200	3.61	25	3.61	150	3.61	200	1.5	200
		2	5	4.88	200	4.88	25	4.88	150	4.88	200	2.03	200
		3	5	6.59	200	6.59	25	6.59	150	6.59	200	2.74	200
		4	5	8.89	200	8.89	25	8.89	150	8.89	200	3.7	200
		5	4	12	100	12	12.5	12	75	12	100	5	100
		5	5	12	200	12	25	12	150	12	200	5	200
Carfilzomib	Panobinostat	5	3	30	3.5	30	3.5	30	3.5	30	3.5	30	3.5
		5	4	30	7	30	7	30	7	30	7	30	7
		5	5	30	14	30	14	30	14	30	14	30	14
	Regorafenib	5	4	30	7500	30	7500	30	7500	30	7500	30	2890
		5	5	30	15000	30	15000	30	15000	30	15000	30	3900
	Romidepsin	4	5	15	200	15	25	15	150	15	200	15	200
		5	2	30	25	30	3.13	30	18.8	30	25	30	25
		5	4	30	100	30	12.5	30	75	30	100	30	100
		5	5	30	200	30	25	30	150	30	200	30	200
MK1775	Carfilzomib	5	4	980	15	980	15	980	15	980	15	980	15
		5	5	980	30	980	30	980	30	980	30	980	30
	Gemcitabine	4	5	490	26	490	2600	490	260	490	260	490	260
		5	4	980	13	980	1300	980	130	980	130	980	130
		5	5	980	26	980	2600	980	260	980	260	980	260
	Ixabepilone	4	4	490	50	490	10	490	10	490	50	490	12.5
		4	5	490	100	490	20	490	20	490	100	490	25
		5	1	980	6.25	980	1.25	980	1.25	980	6.25	980	1.56
		5	2	980	12.5	980	2.5	980	2.5	980	12.5	980	3.13
		5	3	980	25	980	5	980	5	980	25	980	6.25
		5	4	980	50	980	10	980	10	980	50	980	12.5
		5	5	980	100	980	20	980	20	980	100	980	25
	Panobinostat	5	1	980	0.875	980	0.875	980	0.875	980	0.875	980	0.875
		5	2	980	1.75	980	1.75	980	1.75	980	1.75	980	1.75
		5	3	980	3.5	980	3.5	980	3.5	980	3.5	980	3.5
		5	4	980	7	980	7	980	7	980	7	980	7
	Regorafenib	5	5	980	15000	980	15000	980	15000	980	15000	980	3900
	Romidepsin	4	4	490	100	490	12.5	490	75	490	100	490	100
		4	5	490	200	490	25	490	150	490	200	490	200
		5	1	980	12.5	980	1.56	980	9.4	980	12.5	980	12.5
		5	2	980	25	980	3.13	980	18.8	980	25	980	25
		5	3	980	50	980	6.25	980	37.5	980	50	980	50
		5	4	980	100	980	12.5	980	75	980	100	980	100
		5	5	980	200	980	25	980	150	980	200	980	200
Romidepsin	Gemcitabine	4	4	100	13	100	1300	75	130	100	130	100	130
		4	5	100	26	100	2600	75	260	100	260	100	260
		5	1	200	1.63	200	163	150	16.3	200	16.3	200	16.3
		5	3	200	6.5	200	650	150	65	200	65	200	65
		5	4	200	13	200	1300	150	130	200	130	200	130
		5	5	200	26	200	2600	150	260	200	260	200	260
	GSK923295A	5	3	200	250	200	250	150	1750	200	1750	200	1750
		5	4	200	500	200	500	150	3500	200	3500	200	3500
		5	5	200	1000	200	1000	150	7000	200	7000	200	7000

Supplemental Table S3. Concentrations of drugs used in the 45 combinations tested across 5 osteosarcoma cell lines.

IC50 and Fold-Of-Potentiation (FOP) Values of Top 6 Combinations									
**Tx1**	**Tx2**	**Tx1 Single IC50 (ng/mL)**	**Tx2 Conc Index**	**Tx1 Combo IC50 (ng/mL)**	**Tx1 FOP**	**Tx2 Single IC50 (ng/mL)**	**Tx1 Conc Index**	**Tx2 Combo IC50 (ng/mL)**	**Tx2 FOP**
**Bortezomib**	**Panobinostat**	*7.45*	**1**	*5.45*	**1.37 ± 0.53**	*11.85*	**1**	*4.42*	**2.68 ± 1.95**
			**2**	*7.72*	**0.96 ± 0.56**		**2**	*2.93*	**4.04 ± 1.95**
			**3**	*5.03*	**1.48 ± 0.56**		**3**	*3.09*	**3.83 ± 1.23**
			**4**	*4.21*	**1.77 ± 0.59**		**4**	*2.71*	**4.37 ± 0.93**
			**5**	*4.35*	**1.71 ± 0.51**		**5**	*2.46*	**4.81 ± 1.29**
**Bortezomib**	**Romidepsin**	*7.45*	**1**	*5.29*	**1.41 ± 0.45**	*20.02*	**1**	*15.96*	**1.25 ± 0.64**
			**2**	*4.73*	**1.57 ± 0.60**		**2**	*8.19*	**2.45 ± 1.01**
			**3**	*4.80*	**1.55 ± 0.52**		**3**	*4.92*	**4.07 ± 2.45**
			**4**	*4.35*	**1.71 ± 0.81**		**4**	*3.77*	**5.31 ± 3.36**
			**5**	*3.02*	**2.46 ± 1.07**		**5**	*2.96*	**6.75 ± 4.51**
**Carfilzomib**	**Panobinostat**	*23.25*	**1**	*17.35*	**1.34 ± 0.65**	*11.85*	**1**	*20.02*	**0.59 ± 0.38**
			**2**	*17.52*	**1.33 ± 0.44**		**2**	*9.98*	**1.19 ± 0.52**
			**3**	*12.62*	**1.84 ± 0.54**		**3**	*9.16*	**1.29 ± 0.27**
			**4**	*9.95*	**2.34 ± 0.66**		**4**	*3.08*	**3.84 ± 0.81**
			**5**	*8.39*	**2.77 ± 0.80**		**5**	*0.91*	**13.00 ± 8.37**
**Carfilzomib**	**Romidepsin**	*23.25*	**1**	*15.19*	**1.53 ± 0.50**	*20.02*	**1**	*15.34*	**1.31 ± 0.73**
			**2**	*10.74*	**2.16 ± 0.66**		**2**	*17.79*	**1.13 ± 0.68**
			**3**	*5.38*	**4.32 ± 1.66**		**3**	*20.75*	**0.96 ± 0.29**
			**4**	*8.74*	**2.66 ± 1.26**		**4**	*13.23*	**1.51 ± 0.53**
			**5**	*6.17*	**3.77 ± 1.69**		**5**	*13.76*	**1.45 ± 0.77**
**MK1775**	**Ixabepilone**	*208.33*	**1**	*243.02*	**0.86 ± 0.24**	*7.59*	**1**	*8.26*	**0.92 ± 0.48**
			**2**	*207.42*	**1.00 ± 0.31**		**2**	*6.02*	**1.26 ± 0.74**
			**3**	*168.10*	**1.24 ± 0.46**		**3**	*5.63*	**1.35 ± 0.75**
			**4**	*167.66*	**1.24 ± 0.53**		**4**	*8.21*	**0.92 ± 0.47**
			**5**	*80.44*	**2.59 ± 1.86**		**5**	*6.00*	**1.26 ± 0.65**
**MK1775**	**Romidepsin**	*208.33*	**1**	*244.68*	**0.85 ± 0.25**	*20.02*	**1**	*12.31*	**1.63 ± 1.00**
			**2**	*251.42*	**0.83 ± 0.24**		**2**	*25.60*	**0.78 ± 0.45**
			**3**	*260.52*	**0.80 ± 0.29**		**3**	*26.31*	**0.76 ± 0.48**
			**4**	*239.76*	**0.87 ± 0.34**		**4**	*42.39*	**0.47 ± 0.22**
			**5**	*155.55*	**1.34 ± 0.61**		**5**	*3.85*	**5.19 ± 5.38**

Supplemental Table S4. IC50 and fold-of-potentiation (FOP) values of bortezomib:romidepsin and bortezomib:panobinostat tested across 5 osteosarcoma cell lines.

Expanded Order of Addition Results																
Agent Name		Order of Addition	Conc (ng/ml)		U2OS		SAOS2		143B		MNNG		MG63		Average ± SEM	
Tx1	Tx2		Tx1	Tx2	FA	*CI*	FA	*CI*	FA	*CI*	FA	*CI*	FA	*CI*	FA	*CI*
**Bortezomib**	**Panobinostat**	**Concurrent**	6.59	14	**0.995**	*0.13*	**0.750**	*1.05*	**0.997**	*0.54*	**0.932**	*0.50*	**0.997**	*0.38*	**0.933 ± 0.029**	*0.50 ± 0.09*
			8.89	7	**0.996**	*0.15*	**0.705**	*0.89*	**1.000**	*0.57*	**0.975**	*0.54*	**0.995**	*0.59*	**0.937 ± 0.035**	*0.55 ± 0.08*
			8.89	14	**0.999**	*0.08*	**0.823**	*0.95*	**1.000**	*0.53*	**0.989**	*0.46*	**0.998**	*0.47*	**0.964 ± 0.021**	*0.50 ± 0.09*
			12	1.75	**0.979**	*0.32*	**0.619**	*0.84*	**1.000**	*0.72*	**0.974**	*0.73*	**0.991**	*0.98*	**0.916 ± 0.046**	*0.74 ± 0.07*
			12	3.5	**0.996**	*0.19*	**0.709**	*0.85*	**1.000**	*0.71*	**0.987**	*0.64*	**0.993**	*0.90*	**0.941 ± 0.037**	*0.68 ± 0.08*
			12	7	**0.999**	*0.11*	**0.857**	*0.75*	**1.000**	*0.69*	**0.991**	*0.60*	**0.997**	*0.68*	**0.971 ± 0.018**	*0.58 ± 0.07*
			12	14	**0.999**	*0.12*	**0.954**	*0.60*	**1.000**	*0.38*	**0.998**	*0.43*	**0.998**	*0.60*	**0.990 ± 0.006**	*0.44 ± 0.08*
**Bortezomib**	**Panobinostat**	**Tx1 → Tx2**	8.89	14	**0.867**	*0.46*	**0.684**	*0.56*	**0.959**	*0.99*	**0.841**	*0.81*	**0.990**	*0.76*	**0.864 ± 0.038**	*0.72 ± 0.06*
			12	7	**0.954**	*0.41*	**0.715**	*0.68*	**0.999**	*0.84*	**0.955**	*0.82*	**0.994**	*0.85*	**0.924 ± 0.032**	*0.73 ± 0.06*
			12	14	**0.951**	*0.42*	**0.782**	*0.64*	**0.997**	*0.94*	**0.950**	*0.85*	**0.994**	*0.88*	**0.935 ± 0.024**	*0.76 ± 0.06*
**Bortezomib**	**Panobinostat**	**Tx2 → Tx1**	6.59	14	**0.984**	*0.13*	**0.937**	*0.42*	**0.949**	*0.50*	**0.780**	*0.56*	**0.880**	*0.54*	**0.891 ± 0.025**	*0.44 ± 0.05*
			8.89	7	**0.968**	*0.20*	**0.921**	*0.38*	**0.987**	*0.48*	**0.850**	*0.58*	**0.744**	*1.26*	**0.868 ± 0.034**	*0.64 ± 0.15*
			8.89	14	**0.995**	*0.09*	**0.970**	*0.32*	**0.993**	*0.43*	**0.895**	*0.51*	**0.936**	*0.46*	**0.948 ± 0.014**	*0.37 ± 0.05*
			12	1.75	**0.811**	*0.56*	**0.721**	*0.61*	**0.982**	*0.70*	**0.839**	*0.79*	**0.810**	*1.26*	**0.819 ± 0.022**	*0.83 ± 0.09*
			12	3.5	**0.939**	*0.33*	**0.897**	*0.43*	**0.990**	*0.60*	**0.889**	*0.68*	**0.817**	*1.26*	**0.890 ± 0.020**	*0.71 ± 0.12*
			12	7	**0.989**	*0.17*	**0.969**	*0.30*	**0.998**	*0.45*	**0.923**	*0.59*	**0.839**	*1.17*	**0.927 ± 0.026**	*0.59 ± 0.15*
			12	14	**0.998**	*0.07*	**0.988**	*0.25*	**0.999**	*0.36*	**0.941**	*0.54*	**0.959**	*0.44*	**0.971 ± 0.010**	*0.34 ± 0.06*
**Bortezomib**	**Romidepsin**	**Concurrent**	3.61	50	**0.808**	*0.45*	**0.926**	*1.15*	**0.926**	*0.88*	**0.613**	*0.81*	**0.973**	*0.42*	**0.831 ± 0.050**	*0.71 ± 0.11*
			3.61	100	**0.926**	*0.33*	**0.969**	*1.29*	**0.978**	*0.89*	**0.791**	*0.69*	**0.991**	*0.30*	**0.918 ± 0.028**	*0.66 ± 0.14*
			3.61	200	**0.970**	*0.26*	**0.981**	*1.84*	**0.986**	*1.14*	**0.878**	*0.68*	**0.996**	*0.24*	**0.954 ± 0.016**	*0.78 ± 0.23*
			4.88	50	**0.922**	*0.29*	**0.930**	*1.15*	**0.993**	*0.61*	**0.777**	*0.66*	**0.993**	*0.37*	**0.905 ± 0.031**	*0.59 ± 0.10*
			4.88	100	**0.971**	*0.22*	**0.972**	*1.25*	**0.997**	*0.61*	**0.876**	*0.60*	**0.997**	*0.29*	**0.954 ± 0.016**	*0.57 ± 0.13*
			4.88	200	**0.989**	*0.16*	**0.981**	*1.85*	**0.998**	*0.70*	**0.941**	*0.53*	**0.997**	*0.27*	**0.977 ± 0.008**	*0.66 ± 0.21*
			6.59	25	**0.897**	*0.36*	**0.798**	*1.28*	**0.990**	*0.72*	**0.787**	*0.72*	**0.983**	*0.66*	**0.878 ± 0.030**	*0.74 ± 0.10*
			6.59	50	**0.975**	*0.21*	**0.938**	*1.15*	**0.999**	*0.55*	**0.913**	*0.58*	**0.995**	*0.44*	**0.958 ± 0.012**	*0.57 ± 0.10*
			6.59	100	**0.992**	*0.15*	**0.977**	*1.21*	**1.000**	*0.49*	**0.962**	*0.50*	**0.996**	*0.40*	**0.982 ± 0.005**	*0.53 ± 0.11*
			6.59	200	**0.998**	*0.10*	**0.989**	*1.48*	**1.000**	*0.58*	**0.988**	*0.39*	**0.998**	*0.31*	**0.994 ± 0.002**	*0.55 ± 0.15*
			8.89	25	**0.968**	*0.29*	**0.860**	*1.13*	**0.999**	*0.67*	**0.946**	*0.65*	**0.986**	*0.82*	**0.949 ± 0.016**	*0.72 ± 0.09*
			8.89	50	**0.994**	*0.17*	**0.970**	*0.85*	**1.000**	*0.57*	**0.978**	*0.54*	**0.996**	*0.57*	**0.985 ± 0.004**	*0.54 ± 0.07*
			8.89	100	**0.997**	*0.13*	**0.991**	*0.82*	**1.000**	*0.58*	**0.994**	*0.42*	**0.998**	*0.46*	**0.995 ± 0.001**	*0.48 ± 0.07*
			8.89	200	**0.999**	*0.09*	**0.996**	*0.97*	**1.000**	*0.64*	**0.998**	*0.34*	**0.998**	*0.45*	**0.998 ± 0.000**	*0.49 ± 0.09*
			12	12.5	**0.960**	*0.40*	**0.746**	*1.17*	**0.999**	*0.86*	**0.968**	*0.77*	**0.983**	*1.18*	**0.933 ± 0.032**	*0.90 ± 0.11*
			12	25	**0.992**	*0.24*	**0.927**	*0.88*	**1.000**	*0.77*	**0.987**	*0.64*	**0.989**	*1.04*	**0.978 ± 0.010**	*0.74 ± 0.11*
			12	50	**0.998**	*0.16*	**0.989**	*0.57*	**1.000**	*0.79*	**0.996**	*0.51*	**0.996**	*0.77*	**0.995 ± 0.001**	*0.58 ± 0.08*
			12	100	**0.999**	*0.12*	**0.998**	*0.43*	**1.000**	*0.79*	**0.998**	*0.45*	**0.998**	*0.62*	**0.998 ± 0.000**	*0.49 ± 0.08*
			12	200	**0.999**	*0.12*	**0.999**	*0.48*	**1.000**	*0.91*	**0.999**	*0.35*	**0.998**	*0.59*	**0.999 ± 0.000**	*0.50 ± 0.09*
**Bortezomib**	**Romidepsin**	**Tx1 → Tx2**	6.59	50	**0.744**	*0.71*	**0.697**	*0.93*	**0.897**	*0.84*	**0.666**	*0.79*	**0.982**	*0.66*	**0.799 ± 0.045**	*0.78 ± 0.04*
			6.59	100	**0.838**	*0.74*	**0.750**	*1.22*	**0.930**	*0.79*	**0.716**	*0.82*	**0.986**	*0.61*	**0.840 ± 0.039**	*0.82 ± 0.07*
			8.89	25	**0.783**	*0.65*	**0.707**	*0.78*	**0.989**	*0.86*	**0.856**	*0.78*	**0.991**	*0.72*	**0.868 ± 0.035**	*0.75 ± 0.03*
			8.89	50	**0.855**	*0.62*	**0.773**	*0.87*	**0.991**	*0.81*	**0.865**	*0.79*	**0.992**	*0.71*	**0.893 ± 0.027**	*0.76 ± 0.03*
			8.89	100	**0.916**	*0.59*	**0.815**	*1.09*	**0.992**	*0.79*	**0.879**	*0.80*	**0.992**	*0.70*	**0.914 ± 0.023**	*0.78 ± 0.06*
			8.89	200	**0.947**	*0.64*	**0.838**	*1.55*	**0.992**	*0.78*	**0.900**	*0.79*	**0.992**	*0.72*	**0.930 ± 0.020**	*0.88 ± 0.11*
			12	12.5	**0.865**	*0.62*	**0.712**	*0.80*	**0.996**	*0.95*	**0.910**	*0.95*	**0.994**	*0.88*	**0.894 ± 0.033**	*0.84 ± 0.05*
			12	25	**0.907**	*0.57*	**0.837**	*0.72*	**1.000**	*0.79*	**0.960**	*0.80*	**0.995**	*0.80*	**0.941 ± 0.019**	*0.74 ± 0.03*
			12	50	**0.946**	*0.50*	**0.884**	*0.74*	**0.999**	*0.82*	**0.961**	*0.80*	**0.995**	*0.81*	**0.957 ± 0.013**	*0.74 ± 0.04*
			12	100	**0.973**	*0.43*	**0.911**	*0.84*	**0.999**	*0.84*	**0.967**	*0.78*	**0.995**	*0.81*	**0.968 ± 0.010**	*0.75 ± 0.06*
			12	200	**0.984**	*0.41*	**0.931**	*0.99*	**0.999**	*0.82*	**0.971**	*0.77*	**0.995**	*0.83*	**0.975 ± 0.008**	*0.77 ± 0.07*
**Bortezomib**	**Romidepsin**	**Tx2 → Tx1**	3.61	50	**0.840**	*0.34*	**0.962**	*0.74*	**0.894**	*0.83*	**0.725**	*0.54*	**0.981**	*0.11*	**0.879 ± 0.034**	*0.47 ± 0.11*
			3.61	100	**0.939**	*0.24*	**0.984**	*0.85*	**0.963**	*0.86*	**0.826**	*0.51*	**0.995**	*0.05*	**0.934 ± 0.022**	*0.46 ± 0.14*
			3.61	200	**0.982**	*0.14*	**0.991**	*1.25*	**0.982**	*1.12*	**0.894**	*0.51*	**0.997**	*0.04*	**0.964 ± 0.014**	*0.56 ± 0.20*
			4.88	25	**0.757**	*0.41*	**0.912**	*0.69*	**0.784**	*0.89*	**0.700**	*0.56*	**0.967**	*0.18*	**0.841 ± 0.036**	*0.51 ± 0.10*
			4.88	50	**0.918**	*0.24*	**0.977**	*0.57*	**0.970**	*0.60*	**0.830**	*0.46*	**0.992**	*0.08*	**0.930 ± 0.022**	*0.36 ± 0.08*
			4.88	100	**0.971**	*0.16*	**0.991**	*0.66*	**0.991**	*0.59*	**0.892**	*0.43*	**0.996**	*0.05*	**0.962 ± 0.014**	*0.35 ± 0.09*
			4.88	200	**0.991**	*0.10*	**0.994**	*0.95*	**0.996**	*0.69*	**0.937**	*0.41*	**0.997**	*0.05*	**0.979 ± 0.008**	*0.40 ± 0.13*
			6.59	25	**0.874**	*0.31*	**0.957**	*0.50*	**0.948**	*0.65*	**0.834**	*0.50*	**0.984**	*0.16*	**0.917 ± 0.021**	*0.40 ± 0.07*
			6.59	50	**0.960**	*0.19*	**0.989**	*0.40*	**0.987**	*0.56*	**0.903**	*0.42*	**0.994**	*0.09*	**0.962 ± 0.013**	*0.31 ± 0.07*
			6.59	100	**0.988**	*0.12*	**0.995**	*0.48*	**0.997**	*0.49*	**0.936**	*0.39*	**0.997**	*0.06*	**0.978 ± 0.008**	*0.28 ± 0.07*
			6.59	200	**0.996**	*0.07*	**0.997**	*0.67*	**0.999**	*0.50*	**0.965**	*0.35*	**0.998**	*0.06*	**0.988 ± 0.005**	*0.30 ± 0.09*
			8.89	12.5	**0.842**	*0.40*	**0.902**	*0.54*	**0.968**	*0.63*	**0.852**	*0.58*	**0.891**	*0.65*	**0.878 ± 0.019**	*0.57 ± 0.06*
			8.89	25	**0.937**	*0.27*	**0.984**	*0.33*	**0.992**	*0.50*	**0.923**	*0.46*	**0.988**	*0.18*	**0.961 ± 0.010**	*0.33 ± 0.05*
			8.89	50	**0.983**	*0.16*	**0.996**	*0.28*	**0.996**	*0.48*	**0.947**	*0.42*	**0.995**	*0.12*	**0.980 ± 0.007**	*0.27 ± 0.05*
			8.89	100	**0.994**	*0.11*	**0.998**	*0.30*	**0.999**	*0.43*	**0.963**	*0.39*	**0.997**	*0.09*	**0.987 ± 0.005**	*0.25 ± 0.05*
			8.89	200	**0.998**	*0.06*	**0.998**	*0.57*	**1.000**	*0.42*	**0.978**	*0.34*	**0.997**	*0.09*	**0.993 ± 0.003**	*0.28 ± 0.07*
			12	12.5	**0.925**	*0.37*	**0.952**	*0.45*	**0.990**	*0.64*	**0.919**	*0.60*	**0.896**	*0.83*	**0.924 ± 0.014**	*0.60 ± 0.08*
			12	25	**0.963**	*0.28*	**0.992**	*0.27*	**0.998**	*0.47*	**0.963**	*0.46*	**0.989**	*0.23*	**0.979 ± 0.005**	*0.33 ± 0.04*
			12	50	**0.989**	*0.17*	**0.996**	*0.28*	**1.000**	*0.38*	**0.970**	*0.44*	**0.994**	*0.16*	**0.987 ± 0.004**	*0.27 ± 0.04*
			12	100	**0.997**	*0.09*	**0.999**	*0.29*	**1.000**	*0.39*	**0.977**	*0.41*	**0.996**	*0.13*	**0.992 ± 0.003**	*0.25 ± 0.05*
			12	200	**0.999**	*0.07*	**0.998**	*0.51*	**1.000**	*0.39*	**0.986**	*0.36*	**0.997**	*0.11*	**0.995 ± 0.002**	*0.27 ± 0.06*
**Carfilzomib**	**Panobinostat**	**Concurrent**	15	7	**0.948**	*0.22*	**0.881**	*0.67*	**0.928**	*0.90*	**0.716**	*0.60*	**0.980**	*0.55*	**0.875 ± 0.044**	*0.58 ± 0.09*
			15	14	**0.994**	*0.09*	**0.959**	*0.53*	**0.988**	*0.43*	**0.838**	*0.44*	**0.996**	*0.33*	**0.941 ± 0.026**	*0.36 ± 0.06*

			30	0.875	0.872	0.55	0.916	0.83	0.992	0.73	0.853	0.76	0.991	0.86	0.911 ± 0.026	0.76 ± 0.08
			30	1.75	0.926	0.42	0.971	0.64	0.994	0.69	0.883	0.68	0.991	0.87	0.940 ± 0.021	0.68 ± 0.08
			30	3.5	0.974	0.26	0.991	0.45	0.998	0.50	0.922	0.56	0.996	0.68	0.967 ± 0.014	0.51 ± 0.08
			30	7	0.996	0.13	0.998	0.31	1.000	0.39	0.955	0.44	0.998	0.49	0.984 ± 0.008	0.36 ± 0.08
			30	14	1.000	0.05	0.999	0.23	1.000	0.36	0.979	0.33	0.999	0.39	0.993 ± 0.004	0.28 ± 0.07
			30	0.875	0.885	0.54	0.836	1.01	0.981	1.04	0.813	0.85	0.993	0.76	0.888 ± 0.032	0.83 ± 0.09
Carfilzomib	Panobinostat	Tx1 → Tx2	30	1.75	0.927	0.45	0.886	0.90	0.980	1.05	0.792	0.90	0.993	0.77	0.897 ± 0.035	0.81 ± 0.10
			30	3.5	0.949	0.39	0.919	0.81	0.993	0.72	0.812	0.86	0.994	0.72	0.914 ± 0.032	0.70 ± 0.09
			30	7	0.973	0.29	0.949	0.72	0.993	0.74	0.866	0.73	0.996	0.64	0.941 ± 0.023	0.63 ± 0.08
Carfilzomib	Panobinostat	Tx1 → Tx2	30	14	0.988	0.21	0.978	0.54	0.996	0.58	0.909	0.61	0.997	0.62	0.962 ± 0.018	0.52 ± 0.08
			30	14	0.852	0.75	0.997	0.20	0.898	0.53	0.595	0.78	0.805	1.18	0.803 ± 0.049	0.73 ± 0.13
Carfilzomib	Romidepsin	Concurrent	3.75	100	0.832	0.50	0.981	0.98	0.844	1.61	0.690	0.71	0.780	0.89	0.813 ± 0.035	0.93 ± 0.21
			7.5	50	0.833	0.36	0.972	0.72	0.944	0.81	0.649	0.65	0.925	0.48	0.840 ± 0.048	0.59 ± 0.10
			7.5	100	0.927	0.27	0.992	0.66	0.980	0.79	0.792	0.58	0.977	0.32	0.917 ± 0.030	0.51 ± 0.11
			7.5	200	0.974	0.18	0.995	0.88	0.976	1.41	0.868	0.62	0.980	0.34	0.949 ± 0.020	0.65 ± 0.21
			15	25	0.869	0.35	0.985	0.45	0.972	0.75	0.695	0.72	0.981	0.54	0.872 ± 0.050	0.56 ± 0.08
			15	50	0.941	0.25	0.997	0.34	0.997	0.41	0.863	0.48	0.996	0.33	0.944 ± 0.023	0.36 ± 0.05
			15	100	0.980	0.16	0.999	0.27	0.999	0.34	0.925	0.41	0.998	0.26	0.971 ± 0.013	0.29 ± 0.05
			15	200	0.995	0.09	0.999	0.38	1.000	0.35	0.960	0.36	0.999	0.23	0.986 ± 0.007	0.28 ± 0.05
			30	12.5	0.951	0.35	0.995	0.43	0.995	0.70	0.885	0.69	0.991	0.84	0.950 ± 0.021	0.62 ± 0.09
			30	25	0.980	0.24	0.999	0.28	0.999	0.45	0.934	0.54	0.995	0.68	0.972 ± 0.013	0.46 ± 0.09
			30	50	0.986	0.20	0.999	0.27	1.000	0.44	0.975	0.36	0.999	0.47	0.988 ± 0.005	0.36 ± 0.07
			30	100	0.997	0.11	1.000	0.28	1.000	0.43	0.990	0.26	0.999	0.40	0.996 ± 0.002	0.31 ± 0.06
			30	200	0.999	0.08	0.999	0.60	1.000	0.49	0.995	0.21	0.999	0.37	0.998 ± 0.001	0.35 ± 0.07
Carfilzomib	Romidepsin	Tx1 → Tx2	15	100	0.865	0.61	0.742	1.43	0.946	0.79	0.700	0.71	0.968	0.65	0.833 ± 0.044	0.82 ± 0.12
			30	12.5	0.886	0.54	0.933	0.70	0.994	0.74	0.881	0.69	0.995	0.66	0.929 ± 0.020	0.67 ± 0.08
			30	25	0.912	0.49	0.974	0.54	0.999	0.47	0.903	0.64	0.997	0.60	0.949 ± 0.016	0.55 ± 0.07
			30	50	0.937	0.44	0.975	0.52	0.999	0.45	0.919	0.60	0.997	0.60	0.959 ± 0.013	0.53 ± 0.07
			30	100	0.954	0.44	0.978	0.53	0.999	0.44	0.945	0.51	0.996	0.62	0.970 ± 0.010	0.52 ± 0.06
			30	200	0.959	0.53	0.970	0.65	0.996	0.65	0.928	0.61	0.996	0.66	0.965 ± 0.011	0.62 ± 0.08
Carfilzomib	Romidepsin	Tx2 → Tx1	7.5	100	0.793	0.68	0.988	0.72	0.803	1.55	0.642	0.84	0.899	0.37	0.822 ± 0.041	0.79 ± 0.20
			15	50	0.729	0.71	0.993	0.35	0.836	0.94	0.595	0.72	0.934	0.33	0.812 ± 0.050	0.59 ± 0.11
			15	100	0.876	0.52	0.997	0.37	0.957	0.86	0.737	0.67	0.985	0.15	0.898 ± 0.034	0.48 ± 0.12
			15	200	0.949	0.39	0.999	0.48	0.979	1.12	0.830	0.71	0.995	0.09	0.940 ± 0.023	0.51 ± 0.17
			30	12.5	0.675	0.86	0.989	0.32	0.847	0.73	0.609	0.66	0.872	0.91	0.788 ± 0.050	0.71 ± 0.10
			30	25	0.745	0.82	0.997	0.25	0.893	0.77	0.667	0.66	0.963	0.42	0.846 ± 0.045	0.57 ± 0.07
			30	50	0.864	0.65	0.999	0.21	0.975	0.59	0.764	0.61	0.989	0.23	0.905 ± 0.032	0.44 ± 0.07
			30	100	0.936	0.50	1.000	0.18	0.996	0.43	0.848	0.56	0.996	0.14	0.944 ± 0.021	0.34 ± 0.07
			30	200	0.978	0.34	1.000	0.31	0.998	0.50	0.905	0.55	0.997	0.12	0.968 ± 0.013	0.34 ± 0.08
MK1775	Ixabepilone	Concurrent	61.5	40	0.618	0.56	0.967	0.29	0.830	0.41	0.749	0.65	0.870	0.53	0.801 ± 0.039	0.49 ± 0.05
			61.5	80	0.698	0.69	0.969	0.51	0.883	0.53	0.830	0.76	0.886	0.74	0.849 ± 0.030	0.64 ± 0.06
			123	40	0.632	0.67	0.964	0.34	0.877	0.35	0.797	0.65	0.882	0.49	0.824 ± 0.037	0.50 ± 0.06
			123	80	0.680	0.87	0.967	0.57	0.912	0.45	0.872	0.67	0.898	0.66	0.862 ± 0.032	0.64 ± 0.07
			245	20	0.629	0.77	0.950	0.34	0.875	0.33	0.821	0.63	0.859	0.55	0.822 ± 0.036	0.53 ± 0.08
			245	40	0.680	0.79	0.955	0.47	0.919	0.33	0.876	0.58	0.872	0.74	0.857 ± 0.032	0.58 ± 0.09
			245	80	0.706	0.99	0.957	0.77	0.928	0.45	0.896	0.71	0.877	1.18	0.870 ± 0.029	0.82 ± 0.14
			490	20	0.817	0.58	0.952	0.50	0.957	0.30	0.906	0.65	0.860	0.73	0.895 ± 0.017	0.55 ± 0.07
			490	40	0.845	0.55	0.950	0.69	0.962	0.32	0.925	0.62	0.864	1.01	0.907 ± 0.015	0.64 ± 0.11
			490	80	0.866	0.58	0.952	1.00	0.960	0.41	0.931	0.72	0.866	1.49	0.913 ± 0.014	0.84 ± 0.17
			980	5	0.862	0.77	0.942	0.79	0.960	0.49	0.940	0.82	0.864	0.93	0.913 ± 0.014	0.76 ± 0.09
			980	10	0.879	0.69	0.969	0.57	0.971	0.43	0.944	0.79	0.868	0.96	0.925 ± 0.015	0.69 ± 0.08
			980	20	0.906	0.56	0.968	0.64	0.979	0.40	0.951	0.75	0.874	1.04	0.934 ± 0.013	0.68 ± 0.10
			980	40	0.923	0.50	0.970	0.73	0.981	0.39	0.953	0.79	0.875	1.22	0.938 ± 0.012	0.73 ± 0.11
			980	80	0.931	0.50	0.970	0.96	0.977	0.49	0.956	0.85	0.874	1.67	0.940 ± 0.012	0.89 ± 0.17
MK1775	Ixabepilone	Tx1 → Tx2	980	40	0.844	0.87	0.960	0.61	0.967	0.42	0.855	1.53	0.835	1.33	0.869 ± 0.024	0.98 ± 0.19
MK1775	Ixabepilone	Tx2 → Tx1	980	40	0.828	0.59	0.940	0.78	0.951	0.37	0.909	0.70	0.864	0.54	0.895 ± 0.017	0.59 ± 0.09
			980	80	0.865	0.58	0.955	0.98	0.968	0.36	0.926	0.77	0.879	0.75	0.913 ± 0.013	0.69 ± 0.12
MK1775	Romidepsin	Concurrent	61.5	100	0.837	0.45	0.979	0.94	0.769	1.50	0.655	0.90	0.763	0.84	0.793 ± 0.038	0.92 ± 0.17
			123	100	0.883	0.36	0.978	1.00	0.811	1.38	0.689	0.97	0.800	0.73	0.824 ± 0.034	0.89 ± 0.16
			245	50	0.852	0.38	0.961	0.81	0.764	0.98	0.675	1.13	0.802	0.59	0.809 ± 0.034	0.78 ± 0.11
			245	100	0.935	0.24	0.982	0.97	0.861	1.26	0.778	0.92	0.858	0.57	0.879 ± 0.025	0.79 ± 0.15
			490	50	0.935	0.26	0.971	0.79	0.883	0.87	0.848	0.92	0.872	0.56	0.902 ± 0.017	0.68 ± 0.09
			490	100	0.969	0.17	0.986	0.88	0.919	1.12	0.877	0.86	0.904	0.52	0.929 ± 0.015	0.71 ± 0.13
			490	200	0.982	0.14	0.991	1.24	0.931	1.78	0.900	0.86	0.918	0.62	0.943 ± 0.013	0.93 ± 0.24
			980	12.5	0.909	0.54	0.933	1.04	0.958	0.56	0.952	0.70	0.876	0.84	0.926 ± 0.011	0.73 ± 0.09
			980	25	0.953	0.31	0.968	0.82	0.955	0.65	0.949	0.74	0.899	0.73	0.946 ± 0.008	0.65 ± 0.08
			980	50	0.971	0.22	0.986	0.67	0.959	0.79	0.947	0.77	0.617	0.59	0.958 ± 0.007	0.61 ± 0.08
			980	100	0.980	0.18	0.992	0.77	0.968	0.98	0.947	0.81	0.939	0.56	0.964 ± 0.007	0.66 ± 0.10
			980	200	0.987	0.16	0.992	1.28	0.972	1.42	0.953	0.80	0.943	0.64	0.969 ± 0.007	0.86 ± 0.18
MK1775	Romidepsin	Tx1 → Tx2	490	100	0.912	0.48	0.938	0.65	0.956	0.24	0.825	1.01	0.893	0.49	0.881 ± 0.029	0.57 ± 0.13
			490	200	0.921	0.66	0.946	0.76	0.961	0.23	0.865	0.88	0.900	0.48	0.899 ± 0.023	0.59 ± 0.12
			980	100	0.935	0.56	0.985	0.41	0.992	0.23	0.828	1.84	0.917	0.83	0.901 ± 0.036	0.78 ± 0.28
			980	200	0.945	0.65	0.987	0.46	0.993	0.23	0.875	1.46	0.921	0.80	0.921 ± 0.026	0.73 ± 0.22
MK1775	Romidepsin	Tx2 → Tx1	245	50	0.694	0.65	0.954	0.84	0.609	1.30	0.659	0.73	0.671	0.78	0.732 ± 0.038	0.85 ± 0.12

Supplemental Table S5. Order-of-addition results of top 6 two-drug combinations assessed across 5 osteosarcoma cell lines where FA is ≥0.95 in at least one cell line and averaged CI is ≤0.9.

SUPPLEMENTAL LEGENDS

Supplemental Figure S1. Combination screening for drug synergy in osteosarcoma. A, schematic of 5x5 checker-board matrix used for high-throughput screening of drug combinations. B, dose-response curves were obtained for each drug for each cell line. C, synergy calculations were done using the Chou and Talalay combination index (CI), based on the median-effect and mass-action principles. An example of combination screening results (carfilzomib and romidepsin in 143B) showed strong synergism (CI<0.5) with >96% anti-tumor activity for combinations with carfilzomib at 30ng/mL and romidepsin at 12.5 ng/mL, 25 ng/mL, and 50 ng/mL. Results are for concurrent drug applications at 72 hours using CellTiter-Glo viability assay. FA = fraction affected

Supplemental Figure S2. Combination screening results. Mixlow synergy analysis of carfilzomib +panobinostat, carfilzomib+romidepsin, MK1775+ixabepilone, MK1775+romidepsin combinations demonstrating synergy (index<1) at high cytotoxic levels (FA>0.9).

Supplemental Figure S1.

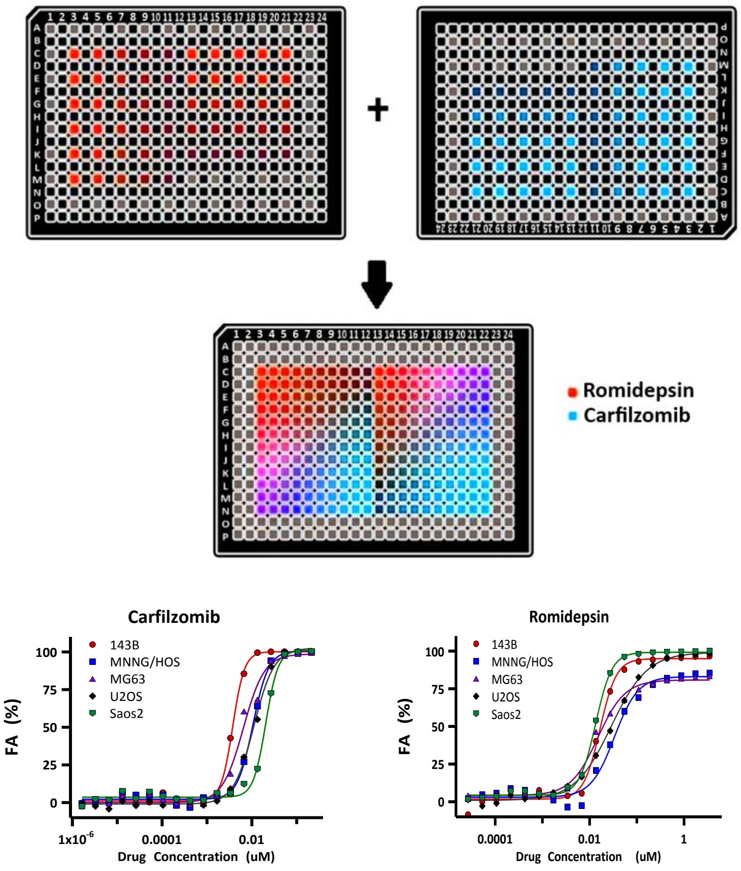

Carfilzomib + Romidepsin

Batch A (19)

Plate 10 - left

FA		Carfilzomib (ng/ml)				
		1.88	3.75	7.5	15	30
Romidepsin (ng/ml)	12.5	0.43	0.48	0.76	**0.96**	**0.99**
	25	0.46	0.51	0.81	**0.96**	**0.99**
	50	0.49	0.57	0.83	**0.96**	**0.99**
	100	0.55	0.61	0.82	**0.96**	**0.99**
	200	0.70	0.71	0.83	**0.89**	**0.97**

CI		Carfilzomib (ng/ml)				
		1.88	3.75	7.5	15	30
Romidepsin (ng/ml)	12.5	1.25	1.2	**0.597**	**0.414**	**0.482**
	25	2.03	1.77	**0.598**	**0.432**	**0.383**
	50	3.27	2.43	**0.74**	**0.432**	**0.398**
	100	4.78	3.71	1.24	**0.519**	**0.464**
	200	4.21	4.06	1.86	1.39	**0.785**

FA vs CI Plot: Carfilzomib x Romidepsin

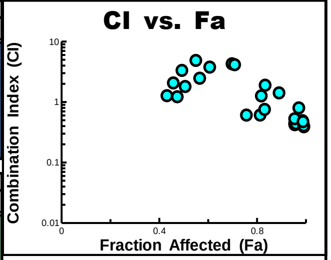

log FA/FU		LOG [Carfilzomib (ng/ml)]				
		0.274	0.574	0.875	1.18	1.48
LOG[Romi (ng/ml)]	1.1	-0.12	-0.04	0.50	1.34	1.91
	1.4	-0.07	0.01	0.64	1.33	2.17
	1.7	-0.01	0.12	0.70	1.39	2.14
	2	0.09	0.19	0.66	1.33	1.99
	2.3	0.37	0.39	0.70	0.92	1.55

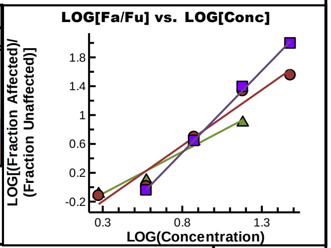

Constant ratio CI values

**Diag 1**	**Dm=** 2.38	**m=** 1.161	**r=** 0.983		**ratio=** 3:40
	**D80=** 7.86	**D85=** 10.61	**D90=** 15.81	**D95=** 30.1	rank= --
	**CI(80)=** *1.4742*	**CI(85)=** *1.4015*	**CI(90)=** *1.3556*	**CI(95)=** 1.4037	CI(avrg)= **1.4088**
**Diag 2**	**Dm=** 2.67	**m=** 1.545	**r=** 0.979		**ratio=** 3:20
	**D80=** 6.54	**D85=** 8.2	**D90=** 11.06	**D95=** 17.94	rank= ++
	**CI(80)=** *0.7812*	**CI(85)=** *0.7250*	**CI(90)=** *0.6815*	**CI(95)=** 0.6704	CI(avrg)= **0.7145**
**Diag 3**	**Dm=** 3.87	**m=** 2.268	**r=** 0.999		**ratio=** 3:10
	**D80=** 7.14	**D85=** 8.32	**D90=** 10.21	**D95=** 14.19	rank= +++
	**CI(80)=** *0.6092*	**CI(85)=** *0.5546*	**CI(90)=** *0.5058*	**CI(95)=** 0.4645	CI(avrg)= **0.5335**

Supplemental Figure S2.

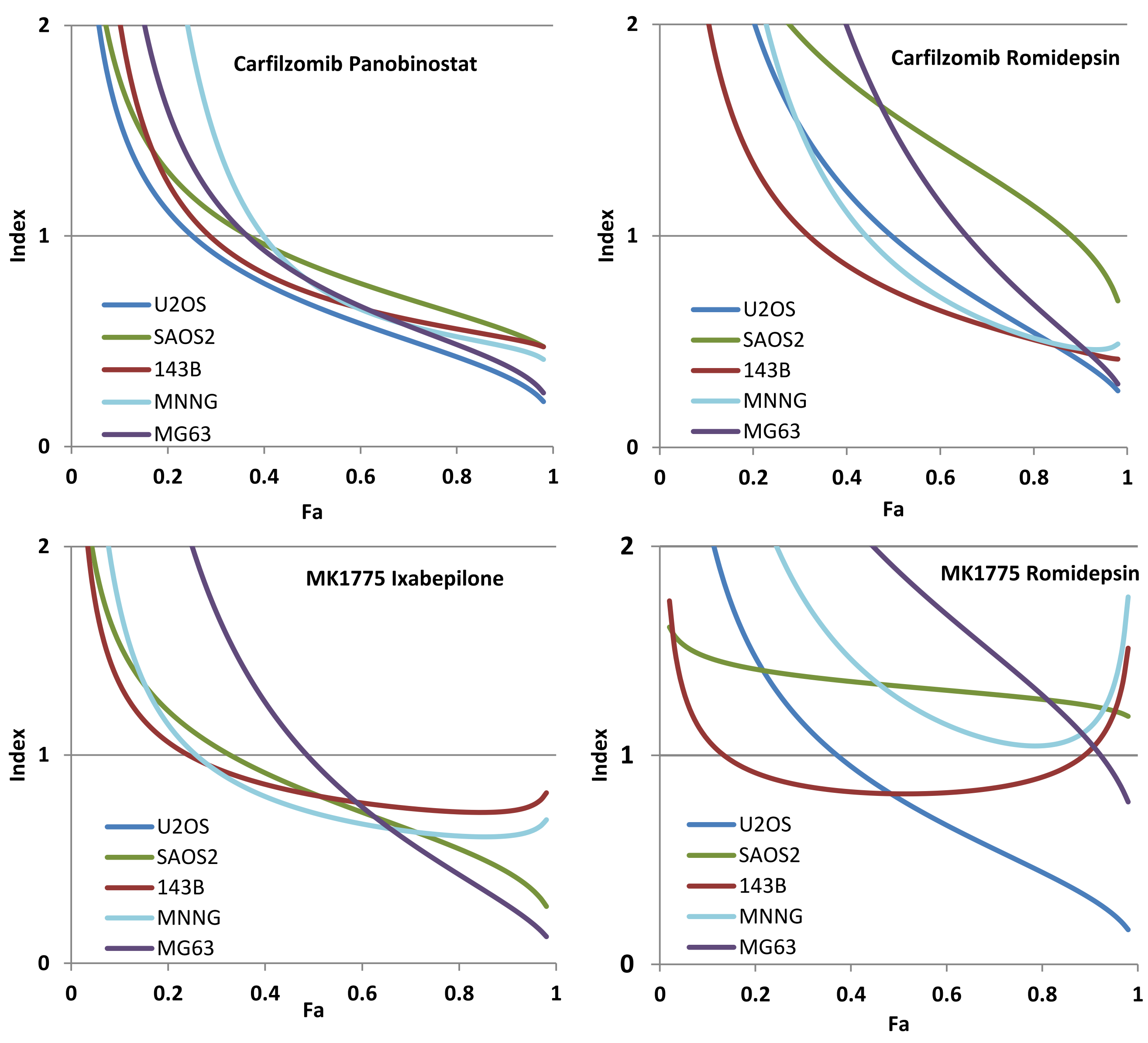

Supplement: Supplementary Information [file srep16991-s1.pdf]
